# Acceptability and Usability of a Theory-Driven Intervention via Email to Promote Physical Activity in Women Who Are Overweight or Obese: Substudy Within a Randomized Controlled Trial

**DOI:** 10.2196/48301

**Published:** 2023-10-03

**Authors:** Jennifer Brunet, Sitara Sharma, Jenson Price, Melissa Black

**Affiliations:** 1 School of Human Kinetics University of Ottawa Ottawa, ON Canada

**Keywords:** behavior change, motivation, overweight, obese, physical activity, women, digital, randomized trial, mobile phone

## Abstract

**Background:**

Insufficient physical activity (PA) and excess weight increase illness risk for women. Face-to-face interventions can increase PA levels; however, they are often inaccessible. With growing interest in digital interventions, a Self-Determination Theory (SDT)–driven intervention was developed and delivered via email to promote PA in women who were insufficiently active and overweight or obese.

**Objective:**

This substudy explores users’ perspectives about the acceptability and usability of the intervention, which was coupled with a wearable activity monitor and PA recommendations.

**Methods:**

A 3-arm, parallel group, randomized controlled trial (unblinded) was conducted in Ontario, Canada. Recruitment occurred from September 2018 to March 2019 via advertising through social media, web-based boards, and posters in publicly accessible areas. In total, 47 women with a BMI of ≥25 kg/m^2^ who were *not* meeting the Canadian PA guidelines were randomly assigned to 1 of 3 arms (arm 1: n=15, 32%; arm 2: n=16, 34%; arm 3: n=16, 34%). This substudy focused on the 15 participants allocated to the main intervention arm. Participants received an automated intervention consisting of (1) 6 weekly emails, (2) a Polar Electro Inc A300 activity monitor (with access to the Polar Flow website and companion smartphone app), and (3) a copy of the Canadian PA guidelines for adults. Emails were developed using SDT and designed to enhance autonomous motivation by fostering the psychological needs of competence, autonomy, and relatedness. Well-established motivational and behavior change techniques were embedded in the emails to promote needs satisfaction. After the intervention (ie, 7 weeks after randomization), participants were invited to complete a web-based acceptability and usability survey containing open-ended and closed-ended questions; responses were analyzed using descriptive and content analyses, respectively.

**Results:**

The analyses included data from 93% (14/15) of the women (age: mean 33.4, SD 7.5; range 24-44 years; BMI: mean 31.3, SD 5.8 kg/m^2^; range 25-40.5 kg/m^2^) who received the main intervention and completed the postintervention survey. Open-ended responses indicated that participants were generally satisfied with the intervention and appreciated that emails prompted self-reflection, kept them on track and accountable, provided informational support, and were nonpressuring. Furthermore, they suggested that the monitor was “enjoyable” and “helpful”; quantitative data corroborated this, as 71% (10/14) said that the monitor was “very valuable/absolutely valuable,” 71% (10/14) would “very probably/definitely” still use one, and 86% (12/14) wore it for ≥5 days per week for ≥8 hours per day and checked it “occasionally/frequently/very frequently.” Potential threats to acceptability included “long” and “text-heavy” emails; lack of personal contact; and cumbersome, unaesthetic monitors.

**Conclusions:**

Results suggest that this SDT-driven, email-delivered intervention may be an acceptable low-contact approach to promote PA in women who are overweight or obese and insufficiently active; however, improvements are warranted and studies ascertaining its effectiveness are needed.

**Trial Registration:**

ClinicalTrials.gov NCT03601663; http://clinicaltrials.gov/ct2/show/NCT03601663

**International Registered Report Identifier (IRRID):**

RR2-10.1177/20552076221093134

## Introduction

### Background

Regular participation in physical activity (PA), a cornerstone of disease prevention, is considered to be beneficial for both physical and psychological health [1]. The Canadian PA guidelines for adults aged 18-64 years [2] recommend engaging in 150 minutes per week of moderate to vigorous intensity PA (MVPA). However, despite the well-documented health benefits of PA [1], general adherence to guidelines is modest [3,4]. The most recent *Canadian Health Measures Survey* (N=2372; age range 18-79 years) revealed that <40% of adults were meeting the Canadian PA guidelines based on objectively measured PA [3]. In addition, studies in Canada [3] and around the world [4] indicate that women, especially those who are overweight or obese (ie, BMI≥25 kg/m^2^), are less likely to adhere to PA guidelines than men, posing a significant threat to women’s long-term health. As increasing PA levels, even without weight change, can improve physical health and attenuate excess weight–related health risks [5], the development of interventions to increase PA levels has been the subject of substantial research. Although increasing women’s awareness of PA guidelines is important, so that they have a target amount in mind, awareness of PA guidelines alone is insufficient to change PA behavior [6]. Rather, helping women increase their PA levels requires consideration of key modifiable determining factors and selection of intervention strategies that will affect change in the determinants.

Health behavior determinants are articulated and explained in numerous theories and models (eg, Self-Determination Theory [SDT] [7], Health Belief Model [8], Theory of Planned Behavior [9], Social Cognitive Theory [10], and the Transtheoretical Model [11]); in turn, they have been used to guide the development and evaluation of interventions intended to increase PA levels (and PA determinants). Current studies suggest that theory-*informed* (ie, those vaguely describing theory use) and theory-*driven* (ie, those integrating theory throughout intervention planning, design, and evaluation; [12]) interventions (henceforth, collectively referred to as “theory based”) may be more effective in increasing PA levels than those that are not theory based [13]. SDT, a major psychological theory of human motivation [7], has been increasingly popular in PA research, and SDT-based interventions have shown superiority in increasing PA levels when compared with usual care [14]. Given that women with a higher BMI who are insufficiently active often display less autonomous motivation to be active [15] and are thus less likely to engage in PA, SDT may be *especially* helpful for increasing PA in this cohort.

Currently, SDT has broad support for producing meaningful behavior change in a variety of health settings, including improving weight management behaviors in women who are overweight or obese [16]. Consistent with SDT, studies show that motivation to engage in PA varies in the degree to which it is experienced as autonomous (or self-determined), with greater autonomous motivation being associated with greater PA [14]. Accordingly, SDT-based interventions aim to foster autonomous motivation to promote behavior change consistent with one’s personal values [14], and many embed strategies to help enhance individuals’ perceptions about competence, autonomy, and relatedness, to increase autonomous motivation while decreasing the controlling sources of motivation. Although there is evidence suggesting that SDT-based PA behavior change interventions offered to women are effective in increasing PA levels [16-19], evidence remains limited regarding intervention acceptability, and caution should be taken when generalizing the existing findings owing to variability in the interventions (ie, content and dosage), technology components, and samples [20]. Thus, additional studies are warranted to design and evaluate acceptable interventions.

The extent to which interventions are acceptable to women may depend on whether the approach to delivery addresses potential challenges they might face with respect to intervention participation. Women have reported multiple and complex role demands and stressors [21] and barriers to PA (eg, lack of time, motivation, and family support; caregiving responsibilities; climate; and safety concerns [22,23]) that can impair their ability to access and engage in behavior change interventions [24]. Practical ways of promoting PA that are effective, efficient, and sustainable should be used to address such barriers. Considering that improvements in infrastructure have allowed a greater number of adults to access the internet, technology-based interventions have emerged as a formidable solution to key barriers that women face and have garnered growing interest as a low-cost means of encouraging PA behavior change [25,26].

Technology-based approaches for promoting PA have included the use of mobile apps, wearable devices, computer software, social media, game-based methods (eg, exergames, virtual reality games, and active video games), emails, and websites to help users address or change PA behaviors, cognitions, and/or psychosocial states [25-27]. Technology-based interventions have shown promising results for increasing PA levels in women who are overweight or obese [18,28,29] and may have the potential to transform health care delivery; however, the use of technology as an interventional tool can also present new challenges [30]. For instance, participants (especially those who are less technologically literate) may struggle to adjust to the apps or tools provided, infrastructure issues (eg, unstable internet connectivity) may hinder participation, and limited or lack of in-person interaction may affect engagement in such interventions. These and other challenges highlight the need to investigate the acceptability of technology-based interventions developed to increase PA levels. This said, studies evaluating the extent to which users consider it to be appropriate, based on anticipated or experiential cognitive and emotional responses to the intervention, are critical [31]. This is important because interventions that are unacceptable will either not be adopted or, if adopted, will not be implemented with fidelity and likely be ineffective. Guidelines for intervention design stipulate the need to consider the acceptability of the intervention from the outset and suggest pretesting the intervention with the target population before full-scale implementation [32]. In addition, recent frameworks for developing and evaluating complex health interventions emphasize a need to focus on their initial development to ensure that they attain the desired change in real-world contexts [33].

### Study Objective

The key to developing effective behavior change interventions is the confirmation that target users consider them acceptable. This substudy evaluated target users’ perspectives about the acceptability and usability of a technology-based intervention driven by SDT that used wearable devices and emails to promote PA in women who were overweight or obese (ie, BMI≥25 kg/m^2^) and insufficiently active (ie, not currently meeting the Canadian PA guidelines for adults aged 18-64 years [2]). Specifically, it aimed to understand users’ perspectives regarding the wearable device, weekly behavioral support emails, and their use patterns. Participants in this substudy were a group of women who were part of a larger randomized controlled trial addressing a different objective (ie, to determine whether adding behavioral support emails to a wearable activity tracker intervention can further increase PA levels in women who are overweight or obese in comparison with a low-contact (ie, wearable activity tracker–only) intervention and a control condition) [34]. Results corresponding to the *effect* of the SDT-driven, email-delivered intervention (ie, the main intervention of interest) on the primary outcome measure of PA are reported elsewhere [35].

## Methods

### Aim, Study Design, and Study Setting

This substudy explored the acceptability of a theory-driven, email-delivered intervention within the context of a larger, single-center, unblinded, 3-arm, parallel group randomized controlled trial [34]. The effects of the intervention were compared with those of a low-contact (ie, wearable activity tracker–only) intervention and a control condition to promote PA in women who were overweight or obese and insufficiently active (refer to Table 1 for an overview of the 3 arms and to the paper by Black and Brunet [34] for detailed information about the study design and protocol). The objectives of the larger trial were to (1) assess changes in PA within each group and determine whether there were significant differences in changes in PA levels between groups and (2) explore changes in PA-related basic psychological needs satisfaction and motivational regulations within and between groups to gain more insight into any observed change (or lack thereof) in PA. As previously reported [34,35], women randomized to the main intervention reported significant increase in walking and in perceptions about competence and relatedness but not in MVPA or in perceptions about autonomy and motivation. Participants’ responses could have been influenced by their subjective perceptions about the intervention and their measurable sustained engagement with the intervention. Thus, to gain insights into which aspects were preferred by participants and which may have limited their motivation to engage with it, participants’ acceptability of the intervention and usability of its components were assessed retrospectively (ie, after they engaged with it) to determine if the intervention content and mode of delivery require modification. The results reported in this paper are presented in accordance with the CONSORT (Consolidated Standards of Reporting Trials) 2010 statement [36], and the CONSORT guidelines for eHealth interventions [37].

**Table 1:** Overview of the trial arms.

Arm^a^	Intervention features		
	Weekly behavioral support emails	Wearable activity tracker with access to the Polar Flow website and companion smartphone app	Basic education on the Canadian physical activity guidelines
Main intervention	Yes	Yes	Yes
Low-contact intervention	No	Yes	Yes
Control condition	No	No	Yes

^a^Refer to the paper by Black and Brunet [34] for further description of each arm.

### Sampling and Recruitment

As previously reported [34], the sample size for the trial was determined a priori based on the primary objective (ie, to determine whether there were significant within-group changes in PA and significant between-group differences in change in PA). As the objective of this substudy was to explore participants’ acceptability of the theory-driven, email-delivered intervention, only those assigned to the main intervention arm were included in this paper.

Participants were recruited for the larger trial from September 2018 to March 2019 via convenience sampling, social media (eg, Facebook), web-based bulletin boards (eg, Kijiji, Craigslist, and local classifieds), and posters in publicly accessible areas (eg, community centers and physicians’ offices). The inclusion criteria were the following: (1) self-identify as female, (2) aged 18-65 years, (3) BMI of ≥25 kg/m^2^, (4) able to read and write in English, (5) report engaging in <150 minutes per week of MVPA and <2 bouts per week of strength or resistance training (eg, free weights, weight machines, resistance bands, and exercises using body weight), (6) have access to the internet and an active email account, and (7) living <50 km from the University of Ottawa. Of note, computer and internet literacy was assumed (and training was provided on the Polar Flow website and companion smartphone app by MB at baseline). Exclusion criteria were the following: (1) be pregnant or lactating, (2) answer “yes” to the question, “do you have any health concerns that could prevent you from safely engaging in physical activity,” and (3) currently use a wearable activity tracking device or have used one in the past 12 months.

### Procedures

Black and Brunet [34] described the protocol for the larger trial in detail. A rolling recruitment strategy was used, wherein each person who contacted MB began their flow through the trial. MB first provided the trial information and assessed interested volunteers for eligibility. Once the eligibility criteria were confirmed, interested volunteers were sent an email that included a link to access a web-based survey platform (ie, SurveyMonkey [SurveyMonkey Inc]). Upon accessing the platform, they were required to read and digitally sign a consent form, after which they were redirected to complete the self-reported measures. Participants then attended a meeting with MB at a convenient location (eg, participants’ homes or university premises) to complete the objective measures (ie, anthropometrics). Once baseline (ie, assessment at week 0) self-report and objective measures were completed, participants were randomized to 1 of 3 arms as described in the *Randomization Procedures* section. Those randomized to the main intervention arm received the first weekly email 1 day following their baseline meeting with MB; subsequent emails were sent to them at 1-week intervals following the first weekly email. Of note, MB encouraged participants to read the emails in sequence over the 6-week intervention period; however, all emails remained accessible (ie, to serve as a resource that participants could return to at any time), which was a key design feature to enhance the accessibility of the intervention. Beyond the unidirectional provision of weekly emails, there was no contact between the participants and researchers during the 6-week intervention period.

In addition to baseline measures, data were collected at 2 other time points, namely, after the intervention (7 weeks after randomization) and at follow-up (21 weeks after randomization). Specifically, 1 day after the sixth email was sent, participants received an email with a link to SurveyMonkey to complete the postintervention self-report measures and were invited to schedule a second in-person meeting with MB to complete the objective measures. Participants (including dropouts) were advised that their data were still valuable regardless of their level of PA participation to promote retention and data collection completeness. Then, 21 weeks after their baseline meeting with MB, participants received another email with a link to SurveyMonkey to complete the follow-up self-report measures. After the intervention and at follow-up, 2 email reminders were sent to nonresponders, 1 and 2 weeks after the initial request.

### Randomization Procedures

Participants were allocated using permuted blocks of 3 and 6 via a web-based randomization software program (Sealed Envelope Ltd; 2017). Randomization was conducted by an independent researcher who was not involved in the trial, and arm allocation was subsequently conveyed to the participants by MB via email. Owing to the nature of the arms and the researchers’ active role in delivering the intervention, participants and researchers were aware of the allocated arm (ie, neither were blinded after assignment to arms). To minimize bias, self-reported measures were administered via the web.

### Intervention Materials

The intervention was developed by MB (ie, a graduate student in the School of Human Kinetics at the University of Ottawa) and her supervisor, JB (ie, a full professor in the School of Human Kinetics at the University of Ottawa). It was designed to be self-guided, so that women could access resources at any time and in any location. It comprised 3 components: (1) a paper copy and brief verbal explanation of the Canadian PA guidelines for adults aged 18-64 years to establish a target for their behavior change, (2) a Polar Electro Inc A300 activity monitor with a charging cable and access to the Polar Flow website and companion smartphone app, and (3) 6 weekly behavioral support emails designed to enhance autonomous motivation for PA. *Component*s *1* and *2* were provided to the participants during their baseline meeting with MB. In light of evidence suggesting that self-monitoring (often conducted using technology) is correlated with PA behavior change [38-40], participants received a wearable activity tracker to self-monitor the frequency and intensity of their PA behavior and their progress toward daily or long-term goals (eg, walking a certain distance over time). They were instructed to wear the device on their wrist daily during waking hours for the 6-week intervention period, except when swimming or bathing. MB provided instructions about how to navigate the device and assisted participants in syncing the device to their computer or smartphone, so that they could review their PA data in greater detail on either the Polar Flow website or its smartphone app. Participants had access to the device, Polar Flow website, and companion smartphone app for the duration of the 6-week intervention and after returning the device to MB at the meeting following the intervention.

*Component 3* featured 6 weekly, nontailored behavioral support emails (ie, the same content was provided to all participants, but emails were addressed to participants, and participants could tailor the content). This modality was chosen as emails are inexpensive, easy to administer, can offer continuous or brief support, and are commonplace in real-world settings. The frequency of emails was set to 1 per week for 6 weeks to strike a balance in providing sufficient support without becoming bothersome. The email content, which featured textual information and worksheets that participants could download and print, was developed by drawing on SDT principles. Accordingly, emails were written using autonomy-supportive phrasing, that is, noncontrolling language to enhance participants’ perceptions about autonomy (ie, perceived control over one’s actions), competence (ie, perceived mastery of tasks and skills), and relatedness (ie, perceived belongingness and connection to others), as well as their autonomous motivation for PA [41,42]. Key motivational and behavior change techniques were selected and embedded into the emails on the basis of previous studies and their relation to SDT [20,43,44]. They aimed to (1) provide information (eg, basic information about the multiple health benefits of regular PA on physical and mental health and to reduce the risk of chronic disease and guidelines for PA), (2) offer feedback or advice (eg, practical advice for starting and maintaining a regular PA schedule with daily walking suggested as an activity and suggestions for how to best form PA habits and restructure the environment to aid), (3) encourage self-monitoring by teaching ways to track PA behavior and experiences (eg, journal activities, reflections, and monitoring with wearable activity tracker), (4) promote goal setting (eg, support to develop personally meaningful and important goals for PA that are specific, measurable, achievable, relevant, and time bound and encouragement to form action plans and coping plans), (5) foster social support (eg, self-identify sources of support and elicit support from such sources), (6) support self-reward (eg, self-identify rewards to obtain upon reaching their goals for PA), and (7) enhance enjoyment (eg, self-reflect on experiences, increase awareness of how thoughts and feelings can lead to inactivity, and thus consider enjoyable activities). Other recurring themes throughout the emails included learning from trial and error, focusing on making small changes, choosing enjoyable activities, and aligning plans with personal beliefs and values. A detailed overview of the content and techniques included in the emails has been published previously [34].

### Measures

A complete description of the primary (ie, PA behavior) and secondary outcome measures (ie, PA-related basic psychological needs satisfaction and motivational regulations), including *how* and *when* they were used, is available in the paper by Black and Brunet [34]. Results of the analyses performed for primary and secondary outcomes are also reported elsewhere [35]. Pertinent to this substudy, participants self-reported sociodemographic and health information (eg, age, marital status, ethnicity, education, work status, and smoking status) at baseline via a web-based survey housed on SurveyMonkey. Participants’ height (m), body mass (kg), body fat (%), and waist circumference (cm) were measured by MB during the in-person meeting at baseline and again after the intervention. These data were summarized to describe the sample.

After the intervention (week 7), acceptability and use patterns data were collected via a survey on SurveyMonkey. As outlined in Table 2, a total of 3 open-ended questions were used. Specifically, participants were asked what they liked and disliked about the intervention, and they were invited to provide points of improvement. Participants were also asked to rate their likelihood of using the wearable activity tracker in the future and to assess its value using a 5-point Likert scale. Finally, participants were presented with 5 questions (each with a list of possible responses to select from) that asked them to indicate how often they used the wearable activity tracker and checked their data on their computer and smartphone.

**Table 2:** Acceptability measures.

Purpose and questions^a^		Response options
**Acceptability of the intervention overall**		
	What did you like about this intervention?	Open-ended, free-text response
	What did you dislike about this intervention?	Open-ended, free-text response
	In your opinion, what could be done to improve this intervention?	Open-ended, free-text response
**Acceptability of the wearable activity tracker**		
	How likely are you to use a wearable activity tracker in the future?	Definitely not, possibly, probably, very probably, and definitely
	How valuable was having a wearable activity tracker for you?	Not valuable at all, of little value, of average value, very valuable, and absolutely valuable
**Device use patterns**		
	On average, how many days per week did you wear your wearable activity tracker?	7 days per week, 6 days per week, 5 days per week, 4 days per week, 3 days per week, 2 days per week, 1 day per week, and 0 days per week
	On days that you wore your wearable activity tracker, how many hours did you wear it?	>10 hours per day, 10 hours per day, 9 hours per day, 8 hours per day, 7 hours per day, 6 hours per day, <6 hours per day, and I did not wear my wearable activity tracker
	On days that you wore your wearable activity tracker, how often did you check your physical activity data on your device?	Very frequently, frequently, occasionally, rarely, very rarely, and I did not wear my wearable activity tracker
	On days that you wore your wearable activity tracker, how often did you check your physical activity data on your smartphone?	Very frequently, frequently, occasionally, rarely, very rarely, and I did not use the smartphone application
	On days that you wore your wearable activity tracker, how often did you check your physical activity data on your computer?	Very frequently, frequently, occasionally, rarely, very rarely, and I did not use the online application

^a^Exact questions asked for acceptability measures.

### Statistical Analysis

Statistical methods pertaining to primary and secondary outcomes have been described elsewhere [34,35]. Descriptive statistics were used to summarize the sample characteristics and responses to the acceptability questions with fixed response options; SPSS (version 26; IBM Corp) was used to conduct these analyses. Content analysis using a deductive approach [45] was performed by 2 trained graduate students familiar with the trial but not otherwise involved in its development or execution (SS and JP) to generate themes derived from participants’ free-text responses to the open-ended acceptability questions. First, SS and JP read through all textual data to obtain a general understanding. Next, they created a preliminary set of codes based on the acceptability questions and searched for themes independently. Codes (including discrepancies) were then discussed, and JB became involved; she reviewed each theme and all open-ended responses to the survey, and discussions occurred until consensus was reached. Microsoft Word was used to store, code, and analyze open-ended survey responses.

### Ethical Considerations

All study activities were approved by the institutional review board at the University of Ottawa (H-06-18-437). Participation was voluntary; all participants provided informed consent digitally in agreement with the Declaration of Helsinki, had the opportunity to ask questions, and were able to withdraw from the study at any time. All study data were deidentified to ensure privacy and confidentiality. Participants received no financial compensation for participating in this study. Consent for publication was obtained from participants.

## Results

### Sociodemographic and Health Characteristics

Recruitment started in September 2018 and ended in March 2019 when the target sample size was reached, and follow-up assessments were conducted until August 2019; a flow diagram including the number of participants who were randomly assigned, received the intervention, and were analyzed is presented elsewhere [35], as are the reasons for exclusions and losses. A total of 15 participants were randomized to the main intervention arm; of these 15 participants, 14 (93%) provided acceptability data after the intervention (refer to Table 3 for *sample* characteristics; characteristics of *all* participants are presented elsewhere [35]). It was not possible to ascertain the reason or reasons for dropout. The 14 women were aged 24-44 years, and most (n=11, 79%) self-identified as White. There were no reports of adverse events or unanticipated effects during the duration of the trial.

**Table 3:** Sociodemographic and health profile of participants at baseline who provided acceptability data after the intervention (n=14).

Variables		Values
Age (years), mean (SD; range)		33.4 (7.5; 24-44)
BMI (kg/m^2^), mean (SD; range)		31.2 (6.1; 25-40.5)
Body fat (%), mean (SD; range)		41.1 (5.8; 24.2-55)
Waist circumference (cm), mean (SD; range)		97.4 (13.1; 71-141)
**Self-rated health, n (%)**		
	Poor	1 (7)
	Fair	4 (29)
	Good	9 (64)
	Very good	0 (0)
	Excellent	0 (0)
**Smoking status, n (%)**		
	Never smoked	13 (93)
	Previously smoked	1 (7)
	Currently smokes	0 (0)
**Education, n (%)**		
	High school	0 (0)
	Some college or university	0 (0)
	College or university	13 (93)
	Graduate degree	1 (7)
**Employment status, n (%)**		
	Unemployed	2 (14)
	Student	3 (21)
	Part-time worker	2 (14)
	Full-time worker	7 (50)
**Annual household income (CAD $ [US $]), n (%)^a^**		
	≤49,999 (36,724)	5 (42)
	50,000-99,999 (36,725-73,449)	3 (25)
	≥100,000 (73,450)	4 (33)
**Race, n (%)**		
	White	11 (79)
	Other	3 (21)

^a^Sample size, n=12 (because 2/14, 14% were not reported).

### Summary of Quantitative Data on Perceived Value and Usability Patterns

Data about perceived value and use of the wearable activity tracker and the likelihood of future use were analyzed descriptively. Scores were generally positive for the value of having a wearable activity tracker; most reported that the tracker was “very valuable” or “absolutely valuable” for them (10/14, 71%) and that they would “very probably” or “definitely” wear it in the future (10/14, 71%). In addition, 71% (10/14) reported wearing the tracker for 6 or 7 days per week, and 100% (14/14) wore it for ≥8 hours per day, with most participants (12/14, 86%) wearing it for ≥12 hours per day. In terms of checking their PA data on the Polar Electro Inc A300 device, 50% (7/14) of the participants did so “very frequently” or “frequently,” whereas the remaining 50% (7/14) checked it on the device “very rarely” or “occasionally.” Similarly, 50% (7/14) “did not use the online app” to check their PA data, 14% (2/14) did so “very rarely,” 21% (3/14) did so “rarely,” and 14% (2/14) did so “occasionally”; none (0/14, 0%) did so “frequently” or “very frequently.” In addition, 29% (4/14) “did not use the smartphone application” to check their PA data, 7% (1/14) did so “very rarely,” 7% (1/14) did so “rarely,” and 43% (6/14) did so “occasionally”; only 14% (2/14) did so “frequently” or “very frequently.”

### Summary of Content Analysis of Open-Ended Responses

#### Overview

The 14 women who completed the postintervention survey provided a total of 41 responses to 3 open-ended acceptability questions (Table 2). The final themes comprise their expressed likes and dislikes pertaining to the intervention and their recommendations for future implementation; these are presented in the following sections, along with quotations from free-text responses to illustrate key ideas. To protect confidentiality, participants were assigned pseudonyms, which are included next to each quotation.

#### Overall Likes Regarding the Intervention and Its Components

Participants’ responses suggested that they were mostly enthusiastic about the email-delivered intervention. Overall, the intervention was considered to be useful because it helped them remain “on track and accountable” (Anna) and offered a sense of accountability in a nonpressuring manner. Participants expressed positive regard for the emails and felt that they were an important element of the intervention. Specifically, they mentioned that the emails provided useful and helpful informational support, offered new ideas and activities that they could try to help meet their PA goals, and helped them learn how to develop feasible or alternative PA goals. Moreover, participants appreciated that the emails contained relevant information, as they were “based around health for women” (Jackie). They also relayed that the information delivered within the emails helped increase their awareness of their PA behavior by prompting self-reflection; a participant highlighted the following:

It got me to think about my actions and where there were problems.

Michelle

Beyond the content, participants said that the frequency of the emails (ie, 1 per week) was appropriate; weekly emails helped keep them engaged and served as “good reminders to reflect on making change” (Peyton). Furthermore, the design and layout of the activity sheets embedded within the emails were appreciated. Finally, participants’ responses demonstrated that they perceived the wearable activity tracker positively, as it offered them an objective measure of their PA; these data were meaningful because it increased participants’ awareness of their current PA levels and helped identify discrepancies between their current and desired levels. The device also served as a visual reminder to participants that their PA levels were being tracked and thus encouraged them to be more active.

#### Overall Dislikes Regarding the Intervention and Its Components

Although the participants largely appreciated the intervention, they also reported dislikes that may have interfered with the acceptability of the intervention and limited their motivation for participation. Their dislikes were related to the style of messaging within the emails (ie, nontailored content), design and delivery of the emails (ie, textual presentation, spacing, timing, and automation), intervention delivery schedule, and device factors. In terms of the style of messaging, participants said that receiving nontailored content was suboptimal because the information provided was not specific to their individual PA needs and preferences; in turn, this reduced their perceived value and utility. A key issue undermining the potential value and utility of the emails was that they only offered “passive guidance” (Maya) and, consequently, did not serve to increase participants’ willingness and capability to successfully engage in PA in their daily lives.

In terms of the design and delivery of the emails, there were 3 specific issues. First, although they could be informative, the emails were automated and therefore did not allow for 2-way communication between the participants and MB. Participants cited 2-way communication as important for ensuring that they engaged with the intervention and felt that having to submit their completed activity sheets would have increased their motivation to complete them. The lack of accountability owing to the 1-way emailing design did little to enhance Priya’s motivation to change her PA behavior, as it was “hard only being accountable to [her]self.” Relatedly, participants disliked that the 1-way emailing did not afford them opportunities to share difficulties, challenges, or failures with MB and receive tailored messaging in return; their responses suggested that this would have been beneficial in preventing negative feelings about not achieving their PA goals. Second, participants felt that the emails lacked examples to visually help them produce their own PA programs, which left them feeling overwhelmed and unsure about how to proceed. Maya explained that “choosing the best activity to make the most noticeable improvement is difficult as there are so many choices of forms of exercise” and she expressed disappointment that the emails “did not lay out example exercise schedules to follow.” Third, some expressed dissatisfaction related to the design (ie, length and density) and delivery (ie, spacing and timing) of the emails. For instance, Leah and Sam described the emails as “long” and “text-heavy,” respectively, whereas Julie explained that she “had trouble completing the weekly self-assessments” because there was insufficient time to do so in-between emails. The time of the week that the emails were sent was also raised as an issue; Jackie suggested that she would have been more inclined to read them had they arrived toward the end of the week as opposed to midweek.

Moreover, the rolling recruitment strategy meant that some participants were recruited during the winter months, which posed challenges. Kristen gave specific reasons for difficulty in engaging in PA (eg, below-freezing temperatures, icy sidewalks, and winter blues) and noted that the timing of the intervention was suboptimal because she had “a really hard time starting new active things outside in the winter.” Finally, participants expressed dissatisfaction with both the physical appearance and accuracy of the Polar Electro Inc A300 activity monitor. They found the monitor to be “cumbersome” and “aesthetically unappealing,” and they worried that it did not register some of their activities, leading to underestimation of their PA levels.

#### Elements of the Intervention That Warrant Reconsideration

The open-ended responses to the survey questions were also valuable in understanding what may help enhance participants’ acceptability of future iterations of the intervention and thus perhaps their motivation, uptake, engagement, and adherence. Although some indicated that there was nothing they would change about the intervention, others made recommendations related to its specific components (ie, emails and device). As a means of improving the emails, participants suggested they be shorter and less text-heavy, and that the embedded activity sheets be revised to contain more background information. In addition, they suggested emails be personalized to provide tailored content for each individual and be delivered when desired by participants. Furthermore, participants suggested adding multimedia components to the emails (eg, video, audio, and graphical illustrations), by “incorporate[ing] some videos” (Anna) and “provid[ing] exercise schedules to use either at home or at a gym” (Peyton). Moreover, in reference to the wearable activity tracker, participants suggested that it be swapped for a device that is more esthetically pleasing and capable of accurately monitoring PA by including a built-in heart rate monitor.

Other recommendations based on participants’ responses related to the overall intervention approach or methods. These included the following: (1) offering a booklet (either digital or print) that collates all the emails and supporting materials to increase accessibility and (2) obtaining greater participant involvement by requesting that activity sheets be returned for review. A participant indicated that “reviewing each week’s assignment with a person who could provide advice and support would help” (Michelle). Participants also desired 2-way communication and suggested the intervention provide both automated (prescheduled) emails *and* opportunities to interact with someone to receive additional support as needed. For the latter aspect, proposed adaptations to the intervention included introducing “a meeting halfway” (Maya), back-and-forth emailing, and phone calls. Others suggested combining the email-delivered intervention with “group or 1-on-1 fitness classes or walks” (Priya); however, participants did not offer opinions about the desired context of such sessions (eg, who should lead them, where they should occur, and how frequently they should be offered).

## Discussion

### Principal Findings

Many researchers have leveraged the strengths of technology to deliver interventions aimed at increasing PA levels in diverse populations, including women [28,38,43,46]. This substudy explored users’ perspectives about the acceptability and usability of a theory-driven intervention delivered via email—a familiar and ubiquitous communication method—that was designed to promote PA in women who were overweight or obese and insufficiently active. Collectively, although derived from a small sample, findings reinforce the critical need for formative studies in the development and delivery of such interventions. Overall, participants provided positive feedback after completing the intervention, suggesting that this type of intervention may meet the specific needs of this cohort. Both quantitative data and open-ended responses helped to understand which aspects of the intervention the participants were satisfied with and used. However, their responses also provided insight into which aspects warrant reconsideration to enhance perceived interest, benefit, enjoyment, utility, and engagement among future users. These aspects must be kept in mind when designing and delivering interventions via email that target women, as the ultimate impact of an intervention depends on engagement, which will be low if women do not find the intervention acceptable.

There is mounting evidence suggesting that self-monitoring is an important interventional strategy for PA behavior change [38,39]. Although various self-monitoring techniques exist (eg, maintaining a PA log or diary), tracking devices and apps are popular [40] because they provide real-time feedback to the user. The quantitative data suggested good overall acceptability and usability of the wearable activity tracker offered to participants, as it was rated as being valuable and likely to be used by most. Correspondingly, participants’ responses to open-ended questions suggested that they valued the objective PA data provided by the tracker because it helped them better understand their current PA levels (or lack thereof) and encouraged them to find ways to engage in more PA throughout their day. However, certain design factors (eg, size and lack of aesthetic appeal) affected the acceptability of the device and participants’ motivation to wear it. The quantitative data about the use patterns of the Polar Flow website and companion smartphone app showed variability between participants; some used these tools more frequently, whereas others used them a limited number of times or not at all. The latter is surprising because the emails included statements that could have prompted more frequent use of the Polar Flow website and companion smartphone app (eg, they encouraged self-monitoring and proposed the website and app as means to do so). Potential reasons for the variation in participants' engagement with these technology-based tools were not queried, but based on a previous study [47], they may include the following: (1) differing preferences for monitoring PA (eg, a preference for printed PA logs), (2) limited opportunities to interact with the tools, (3) feelings of disappointment triggered by the results (eg, failing to reach the step count goal), (4) low perceived utility, (5) increased perceived burden from manual tracking, (6) lack of understanding or trust in the data, making it seem pointless to review its data, and (7) concerns about privacy. In addition, perceived usability or ease of use could have influenced their use patterns [48], that is, previous experience (or lack thereof) in using digital apps, design flaws, and technical issues could also account for the variability in participants’ engagement with the device itself and with the Polar website and companion smartphone app. Unfortunately, there was no interviewer to intervene and ask follow-up questions, which could have given insight into the reasons and offered suggestions to increase use. To fill this knowledge gap, synchronous, in-depth interviewing (either in person, or via telephone or the web) could be used to delve deep into the reasons. Regardless of the reason or reasons behind use or nonuse patterns, it would be helpful to address low device and app use issues in future iterations of this intervention as it is reasonable to expect that it may undermine effectiveness.

SDT offers a framework to target PA determinants, which can then be linked to corresponding intervention strategies or behavior change techniques for optimization. Several studies have used SDT and behavior change techniques to inform their interventions [14,44]. However, the acceptability of email to target SDT constructs and transmit behavior change techniques is uncertain. On the one hand, participants’ responses to the open-ended questions suggest that a series of emails that drew on SDT principles and embedded key motivational and behavior change techniques have the potential to be well received by women who are overweight or obese and insufficiently active because of what they can offer. Specifically, confirming previous behavior change studies [39,49], participants felt that the emails (1) provided useful and helpful informational support, (2) offered new ideas or strategies to meet their PA goals, (3) taught them how to develop feasible or alternative PA goals, and most importantly, (4) prompted self-reflection about their PA behavior, thus motivating them to make the changes necessary to progress toward their personal PA goals. Although there is insufficient evidence to draw conclusions about the effectiveness of *just* the 6 emails, findings provide evidence that SDT-based, email-delivered interventions should be considered to address barriers associated with traditional, face-to-face, health-related interventions (ie, by reducing costs [eg, money and time] and increasing convenience and accessibility [eg, for isolated and stigmatized groups]) [50].

In contrast, participants’ responses also shed light on the potential threats to acceptability and point toward what could be improved as issues were raised regarding the emails. The first issue relates to the fully automated (asynchronous) nature of the emails. Although participants were provided with a description of the intervention, their responses suggest that rather than being passive recipients of information via 1-way emailing, they would have preferred the opportunity to actively participate through interactions with MB (eg, via 2-way emails, phone calls, and meetings) or with other women (eg, via PA classes and groups). This finding is unsurprising given women’s desires for support from credible health experts [51] and engaging with other participants during PA interventions [52]. Recent studies among women, for example, found that interactions with and feedback from others (eg, delivery agent) can act as a motivational factor that promotes intervention engagement and behavior change [20,53-56]. Developing tailored interventions that allow women’s needs, wants, and preferences to dictate the level of human involvement (ie, type of support offered, timing and frequency of the support, how it is initiated, and medium used to deliver support) may be necessary to promote experiential cognitive and emotional responses that facilitate PA during and after the intervention. Accordingly, a stepped care model [57] may be a solution, that is, a fully automated intervention could be initiated first, followed by increased human involvement as needed (eg, stepping up the level of provider involvement to more intensive coaching or other forms of support). It is important for future studies to (1) determine if the introduction of this model renders this intervention more effective, (2) explore the potential ramifications of featuring additional components (eg, costs), and (3) ensure that it could be implemented outside a research setting. Arguably, fully automated interventions are beneficial, as they can be initiated by administrative personnel or through preprogrammed email-based software and thus incur few costs [50,58,59]. In contrast, increasing provider involvement or conducting PA classes may have high implementation costs, as they may require more time to educate providers and participants, facilitate engagement, and support users. Thus, the decision to increase support, although perhaps more acceptable and impactful, should be considered in light of available resources. These are important issues to be explored in future studies via cost metrics to gauge potential sustainability.

Additional issues pertaining to the emails relate to their content, format, style or design, and the predetermined, researcher-set delivery schedule. In terms of the content, format, and style or design of the emails, similar to other interventions delivered via email [60], emails were delivered as text-based information; however, there is evidence suggesting that video-tailored feedback leads to greater attention compared with text-tailored feedback [61]. This suggests that emails should be tailored and personalized to the individual in terms of their content and that some text should be replaced or emphasized with multimedia (eg, videos and graphics) to foster better content engagement. In terms of the delivery protocol, although there is evidence supporting the effectiveness of different “dosages” of emails (eg, single email or 1 per week for several weeks) in increasing PA levels [38,46,62], the dosage has been heterogeneous across studies, and the optimal timing and spacing is not known. In this substudy, there were conflicting perspectives, suggesting that a flexible delivery protocol may be key to fostering intervention acceptance and thus ensuring the desired engagement. Considering the extent of the suggested revision, it will be important to conduct a sufficiently powered trial to test the effects of the revised emails (and delivery protocol) on PA levels.

### Implications

This substudy has important implications for research and practice. Although there has been a proliferation of email-delivered interventions to increase PA levels in women [28], few studies have explored how to construct emails designed to promote PA using a participatory approach. In this substudy, the use of open-ended questions along with a web-based platform to collect data revealed that emails were generally an acceptable way of providing support to women, but they were suboptimal in certain regards and require further development and evaluation. On the basis of the current findings, participant-driven recommendations included the following: (1) reducing the length and density of the emails, (2) varying how information is presented within the emails (eg, adding video, audio, and graphical illustrations to text), (3) providing more information about *how* to engage in PA (eg, through sample programs), and (4) compiling email content into a single booklet that can be printed (if desired). Although these data are informative, it remains necessary to directly ask women specific questions about their preferred email content; doing so will help identify the most facilitative versus gratuitous elements, and that information can be used to determine what (if any) content could be removed to reduce the length of emails. Furthermore, as the design of the emails also requires careful consideration, it would be valuable to consult with health marketing and communication experts regarding the most effective ways to present information (eg, multimedia, layout, font, and colors). For example, to increase user engagement, some text could be replaced with multimedia (eg, illustrations or graphics, audio or video such as recorded content from health professionals and past users, or animated information), testimonials, or personal stories. Importantly, all materials presented should be inclusive of diverse women (eg, video should feature women of varying physical traits and from varying backgrounds). Thus, using a participatory approach (ie, involving interested parties [including target users] in intervention design, testing, and evaluation) may yield a series of emails that women consider as more relevant, appealing, comprehensive, appropriate, meaningful, and practical in terms of PA promotion. In turn, the cocreated emails could be introduced in future interventions as a tool purposely designed with the input and voices of women; this may increase women’s willingness to participate and the effectiveness of the intervention.

In addition, the data collected in this substudy suggest that wearable tracker use could be improved by providing women with a lightweight, stylish, and unobtrusive device that has documented validity and reliability. Accordingly, it could help to involve target users at an early stage of the intervention development to decide which wearable activity tracker would be best for them to monitor their PA behavior. Nevertheless, it will be important to explore factors that may limit engagement with the device itself and its associated website and smartphone app to ensure optimal use because the effectiveness of the intervention is likely to depend on full engagement. Although not investigated in this substudy, such factors may be related to psychosocial, behavioral, or sociodemographic characteristics, including previous experience with using web-based programs [63,64].

Furthermore, consistent with other studies [40], the results suggest that the level of human support needs to be increased, at least for some individuals. In line with participants’ recommendations, weekly emails could be complemented by a midintervention touchpoint (via videoconferencing, telephone, and instant messaging) or back-and-forth email communication to provide more personalized instructions, recommendations, feedback, and support. However, some may require even more human support; thus, as suggested previously, it may be best to provide a continuum of human support that encompasses completely human-delivered interventions, partially-guided interventions, and fully automated interventions and consider allowing participants to self-enroll into their preferred intervention. Regardless of which changes are made to the intervention or delivery protocol, a large trial with adequate power to detect statistical significance is warranted to help determine whether the revised intervention and delivery protocol produce meaningful, observable effects on the primary (ie, PA behavior) and secondary outcomes (ie, PA-related psychological needs satisfaction and motivational regulations). Furthermore, an implicit assumption is that making these changes will improve several implementation outcomes (eg, acceptability, adoption, appropriateness, feasibility, and fidelity). Although possible, analyzing the implementation outcomes following the revised intervention is an important area of further studies, as there may be additional “costs of time” for users that cause them to drop out. However, costs of time are theoretical and deserve further analysis, as they may instead increase participants’ motivation and engagement and thus have greater effects on primary and secondary outcomes.

Finally, a critical yet unresolved issue in this substudy is how well participants felt that the intervention and its components helped foster their PA-related psychological needs satisfaction and autonomous motivation. The latter also warrants further investigation as it serves as an indicator of implementation success. Arguably, enhancing participants’ psychological needs satisfaction and autonomous motivation for PA is a necessary precondition for attaining subsequent desired change in PA behavior. Adopting qualitative methods to conduct an in-depth investigation into how the intervention components each promoted or thwarted the process outcomes will help to advance the knowledge about how these key constructs can be fostered in future iterations of this intervention.

### Limitations

This substudy has limitations that could be addressed in future studies. First, convenience sampling was used, and participants self-selected, which may have resulted in selection bias. Participants may have been more interested in changing their PA behavior (and thus more motivated to fulfill the intervention requirements) or more competent with technology than women in the general population. These factors could have influenced their responses and limited the applicability of the results. Second, only data from those who completed the postintervention assessment (14/15, 93%) were analyzed, which might have biased the results. Third, no procedures were put in place to recruit a heterogenous sample based on sociodemographic characteristics, thus limiting the generalizability of the results. Future studies could use a purposive sampling method to recruit women with *specific* sociodemographic characteristics. Fourth, the exploratory nature of this substudy and sample characteristics did not allow for the investigation of sociodemographic factors that may influence how the intervention would be received and acted upon. Fifth, researcher-created measures were used and these were brief and administered after the intervention; thus, important and relevant evidence regarding acceptability may not have been fully captured. Acceptability is dynamic and can be assessed prospectively, concurrently, and retrospectively [31]. In addition, it is a multifaceted concept that includes the following constructs: affective attitude (ie, how an individual feels about the intervention), burden (ie, perceived amount of effort that is required to participate in the intervention), ethics (ie, goodness of fit between the intervention and individual values), coherence (ie, participant understanding of the intervention and how it works), opportunity costs (ie, extent to which benefits, profits, or values must be given up to engage in the intervention), perceived effectiveness (ie, extent to which the intervention is perceived as likely to achieve its purpose), and self-efficacy (ie, confidence to perform the required behaviors) [31]. Depending on *when* acceptability is assessed and *what* constructs are assessed, the evidence of acceptability may be different. In future studies, acceptability evaluations necessitate comprehensive assessment of these different constructs over time. The theoretical framework of acceptability [31] may be appropriate to inform such assessments. Finally, device usability patterns were assessed via self-report, which can be prone to response bias, and no verification of whether participants read the emails and completed the activity sheets that were sent to them was conducted.

### Conclusions

In-person interventions promoting PA often have considerable implementation costs and may be unappealing for women with barriers such as travel, scheduling, and fear of weight-based stigma. Therefore, the development and evaluation of low-cost, technology-based interventions that can provide women with evidence-based information for increasing and sustaining PA deserves more investigation. Recent frameworks for developing and evaluating complex health interventions emphasize a need to focus on their initial development, as many fail to demonstrate effectiveness in real-world contexts [33]. This substudy was conducted to explore users’ perspectives about the acceptability and usability of an SDT-driven, email-delivered intervention designed to promote PA in women who were overweight or obese and insufficiently active. Results provide preliminary support for the acceptability and usability of the intervention; however, issues that could potentially undermine both were reported. Participants noted preferences for 2-way emailing, individualized delivery schedules, and tailoring of content. Designing appealing emails (including multimedia) and selecting stylish devices were also noted as being important. As researchers continue to investigate the effectiveness of technology-based interventions that integrate wearable devices to help increase PA in women who are overweight or obese and insufficiently active (or otherwise), more studies are needed to implement these changes and engage target users from various sociodemographic backgrounds to realize their full potential. Future studies should also collect fidelity data (eg, via email management systems that allow tracking of email delivery and open rates) to ensure that participants are receiving the intervention as intended and that it produces successful outcomes. Finally, studies aimed at testing the associations between each intervention component, implementation outcomes, and PA outcomes are needed.

## Appendix

Multimedia Appendix 1 CONSORT-eHEALTH checklist (V 1.6.1).

## Abbreviations

CONSORT: Consolidated Standards of Reporting Trials
MVPA: moderate to vigorous intensity physical activity
PA: physical activity
SDT: Self-Determination Theory

## Data Availability

The data sets generated and analyzed during this substudy are not publicly available, as participants were assured that their data would be kept private and confidential to the extent permitted by law and that only the research team would have access to the data.
