# Supplementary material for: Acceptability and Usability of a Theory-Driven Intervention via Email to Promote Physical Activity in Women Who Are Overweight or Obese: Substudy Within a Randomized Controlled Trial
Source: JMIR Form Res. 2023 Oct 3;7:e48301. doi: 10.2196/48301 (PMC10582810; doi:10.2196/48301)
Supplement: Multimedia Appendix 1 [file formative_v7i1e48301_app1.pdf]

CONSORT-EHEALTH (V 1.6.1) - Submission/Publication Form

The CONSORT-EHEALTH checklist is intended for authors of randomized trials evaluating web-based and Internet-based applications/interventions, including mobile interventions, electronic games (incl multiplayer games), social media, certain telehealth applications, and other interactive and/or networked electronic applications. Some of the items (e.g. all subitems under item 5 - description of the intervention) may also be applicable for other study designs.

The goal of the CONSORT EHEALTH checklist and guideline is to be
a) a guide for reporting for authors of RCTs,
b) to form a basis for appraisal of an ehealth trial (in terms of validity)

CONSORT-EHEALTH items/subitems are MANDATORY reporting items for studies published in the Journal of Medical Internet Research and other journals / scientific societies endorsing the checklist.

Items numbered 1., 2., 3., 4a., 4b etc are original CONSORT or CONSORT-NPT (non-pharmacologic treatment) items.
Items with Roman numerals (i., ii, iii, iv etc.) are CONSORT-EHEALTH extensions/clarifications.

As the CONSORT-EHEALTH checklist is still considered in a formative stage, we would ask that you also RATE ON A SCALE OF 1-5 how important/useful you feel each item is FOR THE PURPOSE OF THE CHECKLIST and reporting guideline (optional).

Mandatory reporting items are marked with a red *.
In the textboxes, either copy & paste the relevant sections from your manuscript into this form - please include any quotes from your manuscript in QUOTATION MARKS,
or answer directly by providing additional information not in the manuscript, or elaborating on why the item was not relevant for this study.

YOUR ANSWERS WILL BE PUBLISHED AS A SUPPLEMENTARY FILE TO YOUR PUBLICATION IN JMIR AND ARE CONSIDERED PART OF YOUR PUBLICATION (IF ACCEPTED).
Please fill in these questions diligently. Information will not be copyedited, so please use proper spelling and grammar, use correct capitalization, and avoid abbreviations.

DO NOT FORGET TO SAVE AS PDF _AND_ CLICK THE SUBMIT BUTTON SO YOUR ANSWERS ARE IN OUR DATABASE !!!

Citation Suggestion (if you append the pdf as Appendix we suggest to cite this paper in the caption):
Eysenbach G, CONSORT-EHEALTH Group
CONSORT-EHEALTH: Improving and Standardizing Evaluation Reports of Web-based and Mobile Health Interventions
J Med Internet Res 2011;13(4):e126
URL: http://www.jmir.org/2011/4/e126/
doi: 10.2196/jmir.1923
PMID: 22209829

dariena.wrona@gmail.com Switch account
Not shared
Draft saved

* Indicates required question

Your name *
First Last

Jennifer Brunet

Primary Affiliation (short), City, Country *
University of Toronto, Toronto, Canada

University of Ottawa, Ottawa, Canada

Your e-mail address *
abc@gmail.com

jennifer.brunet@uottawa.ca

Title of your manuscript *
Provide the (draft) title of your manuscript.

A theory-driven intervention delivered by email to promote physical activity in women who are overweight or obese: Participants' perspectives on acceptability and usability within the context of a three-arm parallel group randomized controlled trial

Name of your App/Software/Intervention *
If there is a short and a long/alternate name, write the short name first and add the long name in brackets.

Not applicable.

Evaluated Version (if any)
e.g. "V1", "Release 2017-03-01", "Version 2.0.27913"

Version 1

Language(s) *
What language is the intervention/app in? If multiple languages are available, separate by comma (e.g. "English, French")

English

URL of your Intervention Website or App
e.g. a direct link to the mobile app on app in appstore (itunes, Google Play), or URL of the website. If the intervention is a DVD or hardware, you can also link to an Amazon page.

https://flow.polar.com

URL of an image/screenshot (optional)

Your answer

Accessibility *
Can an enduser access the intervention presently?

- ( ) access is free and open
- ( ) access only for special usergroups, not open
- ( ) access is open to everyone, but requires payment/subscription/in-app purchases
- (•) app/intervention no longer accessible
- ( ) Other:

Primary Medical Indication/Disease/Condition *
e.g. "Stress", "Diabetes", or define the target group in brackets after the condition, e.g. "Autism (Parents of children with)", "Alzheimers (Informal Caregivers of)"

Obesity

Primary Outcomes measured in trial *
comma-separated list of primary outcomes reported in the trial

Physical activity

Secondary/other outcomes
Are there any other outcomes the intervention is expected to affect?

Psychological needs satisfaction/frustration, motivational regulations, acceptability, usability

Recommended "Dose" *
What do the instructions for users say on how often the app should be used?

- ( ) Approximately Daily
- ( ) Approximately Weekly
- ( ) Approximately Monthly
- ( ) Approximately Yearly
- (•) "as needed"
- ( ) Other:

Approx. Percentage of Users (starters) still using the app as recommended after 3 months *

- (•) unknown / not evaluated
- ( ) 0-10%
- ( ) 11-20%
- ( ) 21-30%
- ( ) 31-40%
- ( ) 41-50%
- ( ) 51-60%
- ( ) 61-70%
- ( ) 71%-80%
- ( ) 81-90%
- ( ) 91-100%
- ( ) Other:

Overall, was the app/intervention effective? *

- ( ) yes: all primary outcomes were significantly better in intervention group vs control
- (•) partly: SOME primary outcomes were significantly better in intervention group vs control
- ( ) no statistically significant difference between control and intervention
- ( ) potentially harmful: control was significantly better than intervention in one or more outcomes
- ( ) inconclusive: more research is needed
- ( ) Other:

Article Preparation Status/Stage *
At which stage in your article preparation are you currently (at the time you fill in this form)

- ( ) not submitted yet - in early draft status
- ( ) not submitted yet - in late draft status, just before submission
- (•) submitted to a journal but not reviewed yet
- ( ) submitted to a journal and after receiving initial reviewer comments
- ( ) submitted to a journal and accepted, but not published yet
- ( ) published
- ( ) Other:

Journal *
If you already know where you will submit this paper (or if it is already submitted), please provide the journal name (if it is not JMIR, provide the journal name under "other")

- ( ) not submitted yet / unclear where I will submit this
- ( ) Journal of Medical Internet Research (JMIR)
- ( ) JMIR mHealth and UHealth
- ( ) JMIR Serious Games
- ( ) JMIR Mental Health
- ( ) JMIR Public Health
- (•) JMIR Formative Research
- ( ) Other JMIR sister journal
- ( ) Other:

Is this a full powered effectiveness trial or a pilot/feasibility trial? *

- ( ) Pilot/feasibility
- (•) Fully powered

Manuscript tracking number *
If this is a JMIR submission, please provide the manuscript tracking number under "other" (The ms tracking number can be found in the submission acknowledgement email, or when you login as author in JMIR. If the paper is already published in JMIR, then the ms tracking number is the four-digit number at the end of the DOI, to be found at the bottom of each published article in JMIR)

- ( ) no ms number (yet) / not (yet) submitted to / published in JMIR
- (•) Other: 48301

TITLE AND ABSTRACT

1a) TITLE: Identification as a randomized trial in the title

1a) Does your paper address CONSORT item 1a? *
I.e does the title contain the phrase "Randomized Controlled Trial"? (if not, explain the reason under "other")

- (•) yes
- ( ) Other:

1a-i) Identify the mode of delivery in the title
Identify the mode of delivery. Preferably use "web-based" and/or "mobile" and/or "electronic game" in the title. Avoid ambiguous terms like "online", "virtual", "interactive". Use "Internet-based" only if Intervention includes non-web-based Internet components (e.g. email), use "computer-based" or "electronic" only if offline products are used. Use "virtual" only in the context of "virtual reality" (3-D worlds). Use "online" only in the context of "online support groups". Complement or substitute product names with broader terms for the class of products (such as "mobile" or "smart phone" instead of "iphone"), especially if the application runs on different platforms.

	1	2	3	4	5	
subitem not at all important	( )	( )	( )	(•)	( )	essential

Clear selection

Does your paper address subitem 1a-i? *
Copy and paste relevant sections from manuscript title (include quotes in quotation marks "like this" to indicate direct quotes from your manuscript), or elaborate on this item by providing additional information not in the ms, or briefly explain why the item is not applicable/relevant for your study

Item 1a-i is partially applicable for the following study as one of the modes of delivery is a non-web-based Internet component (i.e., email), which is noted in the title "A theory-driven intervention delivered by email to promote physical activity in women who are overweight or obese: Participants' perspectives on acceptability and usability within the context of a three-arm parallel group randomized controlled trial." The other component included in the following trial is the use of the Polar A300 activity.

1a-ii) Non-web-based components or important co-interventions in title
Mention non-web-based components or important co-interventions in title, if any (e.g., "with telephone support").

	1	2	3	4	5	
subitem not at all important	( )	( )	( )	( )	(•)	essential

Clear selection

Does your paper address subitem 1a-ii?
Copy and paste relevant sections from manuscript title (include quotes in quotation marks "like this" to indicate direct quotes from your manuscript), or elaborate on this item by providing additional information not in the ms, or briefly explain why the item is not applicable/relevant for your study

A theory-driven intervention delivered by email to promote physical activity in women who are overweight or obese: Participants' perspectives on acceptability and usability within the context of a three-arm parallel group randomized controlled trial.

1a-iii) Primary condition or target group in the title
Mention primary condition or target group in the title, if any (e.g., "for children with Type I Diabetes") Example: A Web-based and Mobile Intervention with Telephone Support for Children with Type I Diabetes: Randomized Controlled Trial

	1	2	3	4	5	
subitem not at all important	( )	( )	( )	( )	(•)	essential

Clear selection

Does your paper address subitem 1a-iii? *
Copy and paste relevant sections from manuscript title (include quotes in quotation marks "like this" to indicate direct quotes from your manuscript), or elaborate on this item by providing additional information not in the ms, or briefly explain why the item is not applicable/relevant for your study

A theory-driven intervention delivered by email to promote physical activity in women who are overweight or obese

1b) ABSTRACT: Structured summary of trial design, methods, results, and conclusions
NPT extension: Description of experimental treatment, comparator, care providers, centers, and blinding status.

1b-i) Key features/functionalities/components of the intervention and comparator in the METHODS section of the ABSTRACT
Mention key features/functionalities/components of the intervention and comparator in the abstract. If possible, also mention theories and principles used for designing the site. Keep in mind the needs of systematic reviewers and indexers by including important synonyms. (Note: Only report in the abstract what the main paper is reporting. If this information is missing from the main body of text, consider adding it)

	1	2	3	4	5	
subitem not at all important	( )	( )	( )	( )	(•)	essential

Clear selection

Does your paper address subitem 1b-i? *
Copy and paste relevant sections from the manuscript abstract (include quotes in quotation marks "like this" to indicate direct quotes from your manuscript), or elaborate on this item by providing additional information not in the ms, or briefly explain why the item is not applicable/relevant for your study

Methods: In this three-arm parallel group randomized controlled trial (unblinded), participants allocated to the main intervention arm received an automated intervention consisting of: six weekly emails informed by self-determination theory, a Polar A300 activity monitor (with access to the Polar website and companion smartphone application), and a copy of the Canadian physical activity guidelines for adults. Post-intervention, participants completed an acceptability survey online containing open- and closed-ended questions. Descriptive and content analyses were performed for responses to closed- and open-ended questions, respectively.

1b-ii) Level of human involvement in the METHODS section of the ABSTRACT
Clarify the level of human involvement in the abstract, e.g., use phrases like "fully automated" vs. "therapist/nurse/care provider/physician-assisted" (mention number and expertise of providers involved, if any). (Note: Only report in the abstract what the main paper is reporting. If this information is missing from the main body of text, consider adding it)

	1	2	3	4	5	
subitem not at all important	( )	( )	( )	( )	(•)	essential

Clear selection

Does your paper address subitem 1b-ii?
Copy and paste relevant sections from the manuscript abstract (include quotes in quotation marks "like this" to indicate direct quotes from your manuscript), or elaborate on this item by providing additional information not in the ms, or briefly explain why the item is not applicable/relevant for your study

In this three-arm parallel group randomized controlled trial (unblinded), participants allocated to the main intervention arm received an automated intervention consisting of: six weekly emails informed by self-determination theory, a Polar A300 activity monitor (with access to the Polar website and companion smartphone application), and a copy of the Canadian physical activity guidelines for adults.

1b-iii) Open vs. closed, web-based (self-assessment) vs. face-to-face assessments in the METHODS section of the ABSTRACT
Mention how participants were recruited (online vs. offline), e.g., from an open access website or from a clinic or a closed online user group (closed usergroup trial), and clarify if this was a purely web-based trial, or there were face-to-face components (as part of the intervention or for assessment). Clearly say if outcomes were self-assessed through questionnaires (as common in web-based trials). Note: In traditional offline trials, an open trial (open-label trial) is a type of clinical trial in which both the researchers and participants know which treatment is being administered. To avoid confusion, use "blinded" or "unblinded" to indicated the level of blinding instead of "open", as "open" in web-based trials usually refers to "open access" (i.e. participants can self-enrol). (Note: Only report in the abstract what the main paper is reporting. If this information is missing from the main body of text, consider adding it)

	1	2	3	4	5	
subitem not at all important	( )	( )	( )	(•)	( )	essential

Clear selection

Does your paper address subitem 1b-iii?
Copy and paste relevant sections from the manuscript abstract (include quotes in quotation marks "like this" to indicate direct quotes from your manuscript), or elaborate on this item by providing additional information not in the ms, or briefly explain why the item is not applicable/relevant for your study

Post-intervention, participants completed an acceptability survey online containing open- and closed-ended questions.

1b-iv) RESULTS section in abstract must contain use data
Report number of participants enrolled/assessed in each group, the use/uptake of the intervention (e.g., attrition/adherence metrics, use over time, number of logins etc.), in addition to primary/secondary outcomes. (Note: Only report in the abstract what the main paper is reporting. If this information is missing from the main body of text, consider adding it)

	1	2	3	4	5	
subitem not at all important	( )	( )	( )	( )	(•)	essential

Clear selection

Does your paper address subitem 1b-iv?
Copy and paste relevant sections from the manuscript abstract (include quotes in quotation marks "like this" to indicate direct quotes from your manuscript), or elaborate on this item by providing additional information not in the ms, or briefly explain why the item is not applicable/relevant for your study

Data from 14 women (age=18-63 years, mean body mass index=31.3±5.8 kg/m2) who received the main intervention and completed the post-intervention survey were analyzed.

1b-v) CONCLUSIONS/DISCUSSION in abstract for negative trials
Conclusions/Discussions in abstract for negative trials: Discuss the primary outcome - if the trial is negative (primary outcome not changed), and the intervention was not used, discuss whether negative results are attributable to lack of uptake and discuss reasons. (Note: Only report in the abstract what the main paper is reporting. If this information is missing from the main body of text, consider adding it)

	1	2	3	4	5	
subitem not at all important	( )	(•)	( )	( )	( )	essential

Clear selection

Does your paper address subitem 1b-v?
Copy and paste relevant sections from the manuscript abstract (include quotes in quotation marks "like this" to indicate direct quotes from your manuscript), or elaborate on this item by providing additional information not in the ms, or briefly explain why the item is not applicable/relevant for your study

This self-determination theory-driven, email-delivered intervention may be an acceptable low-contact approach to promote physical activity in women who are overweight or obese and insufficiently active, although improvements are warranted and studies ascertaining its effectiveness are needed.

INTRODUCTION

2a) In INTRODUCTION: Scientific background and explanation of rationale

2a-i) Problem and the type of system/solution
Describe the problem and the type of system/solution that is object of the study: intended as stand-alone intervention vs. incorporated in broader health care program? Intended for a particular patient population? Goals of the intervention, e.g., being more cost-effective to other interventions, replace or complement other solutions? (Note: Details about the intervention are provided in "Methods" under 5)

	1	2	3	4	5	
subitem not at all important	( )	( )	( )	( )	(•)	essential

Clear selection

Does your paper address subitem 2a-i? *
Copy and paste relevant sections from the manuscript (include quotes in quotation marks "like this" to indicate direct quotes from your manuscript), or elaborate on this item by providing additional information not in the ms, or briefly explain why the item is not applicable/relevant for your study

Regular participation in physical activity (PA), a cornerstone of disease prevention, is considered beneficial for both physical and psychological health [1]. The Canadian PA guidelines for adults aged 18-64 years [2] recommend engaging in at least 150 minutes of moderate-to-vigorous intensity PA (MVPA) per week. Despite the well-documented health benefits of PA [3,4], general adherence to PA guidelines is modest [5]. Indeed, the most recent Canadian Health Measures Survey (N=2,372; age range=18-79 years) revealed that less than 40% of adults were meeting the Canadian PA guidelines based on objectively-measured PA [5]. Also, research in Canada [5] and around the world [6] indicates that women, especially those who are overweight or obese (i.e., body mass index [BMI] [7] >25 kg/m2), are less likely to adhere to PA guidelines than men, posing a significant threat to women's long-term health. Whilst increasing women's awareness of PA guidelines is important so they have a PA target in mind (i.e., the primary behavioural outcome), it is critical to provide women strategies to increase compliance because the existence of PA guidelines alone is insufficient to change PA behaviour [8].
Although evidence suggests SDT-based PA behaviour change interventions offered to women are effective in increasing PA levels [20,22-24], developing and implementing such interventions requires an understanding of potential challenges that women face with respect to intervention participation. Technology-based interventions have shown promising results for increasing PA in women who are overweight or obese [23,32,33] and may have the potential to transform healthcare delivery; however, the use of technology as an interventional tool can also present new challenges [34].

2a-ii) Scientific background, rationale: What is known about the (type of) system
Scientific background, rationale: What is known about the (type of) system that is the object of the study (be sure to discuss the use of similar systems for other conditions/diagnoses, if appropiate), motivation for the study, i.e. what are the reasons for and what is the context for this specific study, from which stakeholder viewpoint is the study performed, potential impact of findings [2]. Briefly justify the choice of the comparator.

	1	2	3	4	5	
subitem not at all important	( )	( )	( )	( )	(•)	essential

Clear selection

Does your paper address subitem 2a-ii? *
Copy and paste relevant sections from the manuscript (include quotes in quotation marks "like this" to indicate direct quotes from your manuscript), or elaborate on this item by providing additional information not in the ms, or briefly explain why the item is not applicable/relevant for your study

Whilst increasing women's awareness of PA guidelines is important so they have a PA target in mind (i.e., the primary behavioural outcome), it is critical to provide women strategies to increase compliance because the existence of PA guidelines alone is insufficient to change PA behaviour [8]. Indeed, helping women increase their PA behaviour requires consideration of key modifiable determining factors and selection of intervention strategies that will affect change in the determinants.
Health behaviour determinants are articulated and explained in numerous individual-level theories and models (e.g., Health Belief Model [9], Theory of Planned Behaviour [10], Social Cognitive Theory [11], Transtheoretical Model [12]); in turn, they have been used to guide the development and evaluation of interventions intended to increase PA (and its determinants). Current research suggests theory-informed (i.e., those vaguely describing theory use) and theory-driven (i.e., those integrating theory throughout intervention planning, design, and evaluation; [13]) interventions (henceforth collectively referred to as 'theory-based) may be more effective at increasing PA than those that are not theory-based [14]. Among existing theories and models, Self-Determination Theory (SDT; [15]) has been applied often in recent years and SDT-based interventions have shown superiority in increasing PA as compared to usual care [16,17]. Given that women with a higher BMI who are insufficiently active often display less autonomous motivation to be active [18] and are thus less likely to engage in PA for a prolonged period of time [19]. SDT may be especially helpful for increasing PA in this group.
SDT now has broad support for producing meaningful behaviour change in a variety of health settings, including improving weight management behaviours in women who are overweight or obese [20]. Consistent with SDT, research shows motivation to engage in PA varies to the degree to which it is experienced as autonomous (or self-determined), with greater autonomous motivation associated with greater PA [21]. Accordingly, the primary

goal of SDT-based interventions should be to foster autonomous motivation to promote behaviour change consistent with one's personal values [21]. Additionally, interventions should embed strategies to help enhance individuals' perceptions of competence, autonomy, and relatedness to increase autonomous motivation while decreasing controlling sources of motivation.
Although evidence suggests SDT-based PA behaviour change interventions offered to women are effective in increasing PA levels [20,22-24], developing and implementing such interventions requires an understanding of potential challenges that women face with respect to intervention participation. For instance, women have reported multiple and complex role demands and stressors [25] as well as barriers to PA (e.g., lack of time, motivation, family support, caregiving responsibilities, climate, and safety concerns [26,27]) that can impair their ability to access and engage in behaviour change interventions [28]. Thus, these barriers should be considered carefully, and practical ways of promoting PA that are effective, efficient, and sustainable should be utilized. During the past decade, improvements in infrastructure have allowed a greater number of adults to access the Internet, providing a formidable solution to the barriers that women face and garnering growing interest in low-cost technology-based interventions to encourage PA [29,30]. Technology-based approaches for promoting PA have included the use of mobile applications, wearable devices, computer software, social media, game-based methods (e.g., exergames, virtual reality games, active video games), e-mails, and websites to help end-users address or change PA behaviours, cognitions, and/or psychosocial states [29-31]. Technology-based interventions have shown promising results for increasing PA in women who are overweight or obese [23,32,33] and may have the potential to transform healthcare delivery; however, the use of technology as an interventional tool can also present new challenges [34]. For instance, participants (especially those who are less technologically-literate) may struggle to adjust to the applications/tools provided, infrastructure issues (e.g., unstable internet connectivity) may hinder participation, and limited/lack of in-person interaction may affect engagement in such interventions. Thus, there is a need to investigate the acceptability of technology-based interventions developed to increase PA; that is, researchers should evaluate the extent to which those receiving the intervention consider it to be appropriate, based on anticipated or experiential cognitive and emotional responses to the intervention [35]. This is important because interventions that are unacceptable will either not be adopted, or, if adopted, they will not be implemented with fidelity and likely be ineffective. Indeed, guidelines for the design of interventions stipulate the need to consider the acceptability of interventions from the outset and suggest pretesting of the intervention with the target population prior to full-scale implementation [36]. As well, recent frameworks for developing and evaluating complex health interventions emphasize a need to focus on their initial development to ensure they attain desired change in real-world contexts [37].

2b) In INTRODUCTION: Specific objectives or hypotheses

Does your paper address CONSORT subitem 2b? *
Copy and paste relevant sections from the manuscript (include quotes in quotation marks "like this" to indicate direct quotes from your manuscript), or elaborate on this item by providing additional information not in the ms, or briefly explain why the item is not applicable/relevant for your study

The key to developing effective behaviour change interventions requires confirmation that target end-users consider it acceptable. Thus, this study evaluated participants' perspectives of the acceptability and usability of a technology-based intervention driven by SDT that utilized wearable devices and emails to promote PA in women who were overweight or obese (i.e., BMI ≥25 kg/m2) and insufficiently active (i.e., not currently meeting Canadian PA guidelines for adults aged 18-64 years; [2]). Specifically, it aimed to understand participants' perspectives regarding the wearable device and the weekly behaviour support emails, as well as their usage patterns for both. Participants in this study were a group of women who were part of a larger randomized controlled trial (RCT) addressing a different objective (i.e., to determine if adding behavioural support emails to a wearable activity tracker intervention can further increase PA levels in women who are overweight or obese in comparison to a wearable activity tracker–only intervention and a control condition) [7]. Results corresponding to the effect of the SDT-driven, email-delivered intervention (i.e., the main intervention of interest) on the primary outcome measure of PA are reported elsewhere [7].

METHODS

3a) Description of trial design (such as parallel, factorial) including allocation ratio

Does your paper address CONSORT subitem 3a? *
Copy and paste relevant sections from the manuscript (include quotes in quotation marks "like this" to indicate direct quotes from your manuscript), or elaborate on this item by providing additional information not in the ms, or briefly explain why the item is not applicable/relevant for your study

This study explored the acceptability of a theory-driven, email-delivered intervention within the context of a larger, single centre, unblinded, three-arm parallel group RCT. Once baseline (i.e., pre-randomization assessment; week 0) self-report and objective measures were completed, participants were randomized to one of three arms. Participants were allocated using permuted blocks of three and six via a web-based randomization software program (Sealed Envelope Ltd., 2017).

3b) Important changes to methods after trial commencement (such as eligibility criteria), with reasons

Does your paper address CONSORT subitem 3b? *
Copy and paste relevant sections from the manuscript (include quotes in quotation marks "like this" to indicate direct quotes from your manuscript), or elaborate on this item by providing additional information not in the ms, or briefly explain why the item is not applicable/relevant for your study

Item 3b is not applicable for the following study because no changes to methods were needed after trial commencement.

3b-i) Bug fixes, Downtimes, Content Changes
Bug fixes, Downtimes, Content Changes: ehealth systems are often dynamic systems. A description of changes to methods therefore also includes important changes made on the intervention or comparator during the trial (e.g., major bug fixes or changes in the functionality or content) (5-iii) and other "unexpected events" that may have influenced study design such as staff changes, system failures/downtimes, etc. [2].

	1	2	3	4	5	
subitem not at all important	●	○	○	○	○	essential

Clear selection

Does your paper address subitem 3b-i?
Copy and paste relevant sections from the manuscript (include quotes in quotation marks "like this" to indicate direct quotes from your manuscript), or elaborate on this item by providing additional information not in the ms, or briefly explain why the item is not applicable/relevant for your study

Item 3b-i is not applicable for the following study because no Bug fixes, Downtimes, or Content Changes needed to be made on the intervention or comparator during the trial.

4a) Eligibility criteria for participants

Does your paper address CONSORT subitem 4a? *
Copy and paste relevant sections from the manuscript (include quotes in quotation marks "like this" to indicate direct quotes from your manuscript), or elaborate on this item by providing additional information not in the ms, or briefly explain why the item is not applicable/relevant for your study

The inclusion criteria were: (a) self-identify as female, (b) age between 18 and 65 years, (c) BMI ≥25 kg/m2, (d) able to read and write in English, (e) report engaging in <150 minutes of MVPA and strength or resistance training (e.g., free weights, weight machines, resistance bands, exercises using body weight) <2 times per week, (f) have access to the Internet and an active email account, and (g) living <50 km of the University of Ottawa. Of note, computer/Internet literacy was assumed (and training was provided on Polar website and companion smartphone application by MB at baseline). Exclusion criteria were: (a) be pregnant or lactating, (b) answer 'yes' to the question, "do you have any health concerns that could prevent you from safely engaging in physical activity?", and (c) currently use or have used a wearable activity tracking device in the past 12 months.

4a-i) Computer / Internet literacy
Computer / Internet literacy is often an implicit "de facto" eligibility criterion - this should be explicitly clarified.

	1	2	3	4	5	
subitem not at all important	○	○	○	○	●	essential

Clear selection

Does your paper address subitem 4a-i?
Copy and paste relevant sections from the manuscript (include quotes in quotation marks "like this" to indicate direct quotes from your manuscript), or elaborate on this item by providing additional information not in the ms, or briefly explain why the item is not applicable/relevant for your study

Of note, computer/Internet literacy was assumed (and training was provided on Polar website and companion smartphone application by MB at baseline).

4a-ii) Open vs. closed, web-based vs. face-to-face assessments:
Open vs. closed, web-based vs. face-to-face assessments: Mention how participants were recruited (online vs. offline), e.g., from an open access website or from a clinic, and clarify if this was a purely web-based trial, or there were face-to-face components (as part of the intervention or for assessment), i.e., to what degree got the study team to know the participant. In online-only trials, clarify if participants were quasi-anonymous and whether having multiple identities was possible or whether technical or logistical measures (e.g., cookies, email confirmation, phone calls) were used to detect/prevent these.

	1	2	3	4	5	
subitem not at all important	○	○	○	○	●	essential

Clear selection

Does your paper address subitem 4a-ii? *
Copy and paste relevant sections from the manuscript (include quotes in quotation marks "like this" to indicate direct quotes from your manuscript), or elaborate on this item by providing additional information not in the ms, or briefly explain why the item is not applicable/relevant for your study

Participants were recruited for the larger trial via convenience sampling online using social media (i.e., Facebook) and online boards (i.e., Kijiji, Craigslist, local classifieds), and offline using posters in publicly accessible areas (i.e., community centers, physician's offices). Once eligibility criteria were confirmed, persons were sent an email that included a link to access an online survey platform (i.e., SurveyMonkey). Upon accessing the platform, persons were required to read a consent form and digitally sign it, after which they were redirected to complete self-reported measures. Participants then attended a meeting with MB at a convenient location (e.g., participants' homes, university premises) to complete objective measures (i.e., anthropometrics). One day after the last email was sent to participants, they received an email with a link to an online platform to complete self-report measures and were invited to schedule a second in-person meeting with MB to complete objective measures (i.e., post-intervention assessment; week 7). Participants, including dropouts, were advised that their data were still valuable regardless of their level of actual PA participation to promote retention and date collection completeness. Twenty-one weeks after their baseline meeting with MB, participants received a final email with a link to an online platform to complete self-report measures (i.e., follow-up assessment; week 21).

4a-iii) Information giving during recruitment
Information given during recruitment. Specify how participants were briefed for recruitment and in the informed consent procedures (e.g., publish the informed consent documentation as appendix, see also item X26), as this information may have an effect on user self-selection, user expectation and may also bias results.

	1	2	3	4	5	
subitem not at all important	○	○	○	○	●	essential

Clear selection

Does your paper address subitem 4a-iii?
Copy and paste relevant sections from the manuscript (include quotes in quotation marks "like this" to indicate direct quotes from your manuscript), or elaborate on this item by providing additional information not in the ms, or briefly explain why the item is not applicable/relevant for your study

Black and Brunet (38) described the protocol for the larger trial in detail. In short, a rolling recruitment strategy was used wherein each person who contacted MB began their flow through the trial procedures. MB first provided study information and assessed interested persons for eligibility. Once eligibility criteria were confirmed, persons were sent an email that included a link to access an online survey platform (i.e., SurveyMonkey). Upon accessing the platform, persons were required to read a consent form and digitally sign it, after which they were redirected to complete self-reported measures. Participants then attended a meeting with MB at a convenient location (e.g., participants' homes, university premises) to complete objective measures (i.e., anthropometrics). Once baseline (i.e., pre-randomization assessment; week 0) self-report and objective measures were completed, participants were randomized to one of three arms. Those randomized to the main intervention arm received the first weekly email one day following their baseline meeting with MB. Subsequent emails were sent to them at one-week intervals following the first weekly email. Of note, MB encouraged participants to read the emails in sequence over the six-week intervention period; however, all emails remained accessible (i.e., emails became a resource that participants could return to at any time), which was a key design feature to enhance accessibility of the intervention.

4b) Settings and locations where the data were collected

Does your paper address CONSORT subitem 4b? *
Copy and paste relevant sections from the manuscript (include quotes in quotation marks "like this" to indicate direct quotes from your manuscript), or elaborate on this item by providing additional information not in the ms, or briefly explain why the item is not applicable/relevant for your study

Once eligibility criteria were confirmed, persons were sent an email that included a link to access an online survey platform (i.e., SurveyMonkey). Upon accessing the platform, persons were required to read a consent form and digitally sign it, after which they were redirected to complete self-reported measures. Participants then attended a meeting with MB at a convenient location (e.g., participants' homes, university premises) to complete objective measures (i.e., anthropometrics). Once baseline (i.e., pre-randomization assessment; week 0) self-report and objective measures were completed, participants were randomized to one of three arms. Those randomized to the main intervention arm received the first weekly email one day following their baseline meeting with MB. Subsequent emails were sent to them at one-week intervals following the first weekly email. Of note, MB encouraged participants to read the emails in sequence over the six-week intervention period; however, all emails remained accessible (i.e., emails became a resource that participants could return to at any time), which was a key design feature to enhance accessibility of the intervention. Beyond the unidirectional provision of weekly emails, there was no contact between participants and the researchers during the six-week intervention period. One day after the last email was sent to participants, they received an email with a link to an online platform to complete self-report measures and were invited to schedule a second in-person meeting with MB to complete objective measures (i.e., post-intervention assessment; week 7). Participants, including dropouts, were advised that their data were still valuable regardless of their level of actual PA participation to promote retention and date collection completeness. Twenty-one weeks after their baseline meeting with MB, participants received a final email with a link to an online platform to complete self-report measures (i.e., follow-up assessment; week 21). Post-intervention and at follow-up, two email reminders were sent to non-responders at one and two weeks after the initial request.

4b-i) Report if outcomes were (self-)assessed through online questionnaires
Clearly report if outcomes were (self-)assessed through online questionnaires (as common in web-based trials) or otherwise.

	1	2	3	4	5	
subitem not at all important	○	○	○	○	●	essential

Clear selection

Does your paper address subitem 4b-i? *
Copy and paste relevant sections from the manuscript (include quotes in quotation marks "like this" to indicate direct quotes from your manuscript), or elaborate on this item by providing additional information not in the ms, or briefly explain why the item is not applicable/relevant for your study

Once eligibility criteria were confirmed, persons were sent an email that included a link to access an online survey platform (i.e., SurveyMonkey). Upon accessing the platform, persons were required to read a consent form and digitally sign it, after which they were redirected to complete self-reported measures.
One day after the last email was sent to participants, they received an email with a link to an online platform to complete self-report measures and were invited to schedule a second in-person meeting with MB to complete objective measures (i.e., post-intervention assessment; week 7). Participants, including dropouts, were advised that their data were still valuable regardless of their level of actual PA participation to promote retention and date collection completeness. Twenty-one weeks after their baseline meeting with MB, participants received a final email with a link to an online platform to complete self-report measures (i.e., follow-up assessment; week 21).

4b-ii) Report how institutional affiliations are displayed
Report how institutional affiliations are displayed to potential participants [on ehealth media], as affiliations with prestigious hospitals or universities may affect volunteer rates, use, and reactions with regards to an intervention.(Not a required item – describe only if this may bias results)

	1	2	3	4	5	
subitem not at all important	●	○	○	○	○	essential

Clear selection

Does your paper address subitem 4b-ii?
Copy and paste relevant sections from the manuscript (include quotes in quotation marks "like this" to indicate direct quotes from your manuscript), or elaborate on this item by providing additional information not in the ms, or briefly explain why the item is not applicable/relevant for your study

The trial protocol was approved by the Institutional Review Board at the University of Ottawa (H-06-18-437) before recruitment and data collection began.

5) The interventions for each group with sufficient details to allow replication, including how and when they were actually administered

5-i) Mention names, credential, affiliations of the developers, sponsors, and owners
Mention names, credential, affiliations of the developers, sponsors, and owners [6] (if authors/evaluators are owners or developer of the software, this needs to be declared in a "Conflict of interest" section or mentioned elsewhere in the manuscript).

	1	2	3	4	5	
subitem not at all important	○	○	○	○	●	essential

Clear selection

Does your paper address subitem 5-i?
Copy and paste relevant sections from the manuscript (include quotes in quotation marks "like this" to indicate direct quotes from your manuscript), or elaborate on this item by providing additional information not in the ms, or briefly explain why the item is not applicable/relevant for your study

The intervention was developed by MB (i.e., a graduate student in the School of Human Kinetics at the University of Ottawa) and her supervisor, JB (i.e., an Associate Professor in the School of Human Kinetics at the University of Ottawa).

5-ii) Describe the history/development process
Describe the history/development process of the application and previous formative evaluations (e.g., focus groups, usability testing), as these will have an impact on adoption/use rates and help with interpreting results.

	1	2	3	4	5	
subitem not at all important	○	●	○	○	○	essential

Clear selection

Does your paper address subitem 5-ii?
Copy and paste relevant sections from the manuscript (include quotes in quotation marks "like this" to indicate direct quotes from your manuscript), or elaborate on this item by providing additional information not in the ms, or briefly explain why the item is not applicable/relevant for your study

This is the first evaluation of this intervention.

5-iii) Revisions and updating
Revisions and updating. Clearly mention the date and/or version number of the application/intervention (and comparator, if applicable) evaluated, or describe whether the intervention underwent major changes during the evaluation process, or whether the development and/or content was "frozen" during the trial. Describe dynamic components such as news feeds or changing content which may have an impact on the replicability of the intervention (for unexpected events see item 3b).

	1	2	3	4	5	
subitem not at all important	○	○	○	○	●	essential

Clear selection

Does your paper address subitem 5-iii?
Copy and paste relevant sections from the manuscript (include quotes in quotation marks "like this" to indicate direct quotes from your manuscript), or elaborate on this item by providing additional information not in the ms, or briefly explain why the item is not applicable/relevant for your study

The following manuscript presents version 1.

5-iv) Quality assurance methods
Provide information on quality assurance methods to ensure accuracy and quality of information provided [1], if applicable.

	1	2	3	4	5	
subitem not at all important	○	○	○	○	●	essential

Clear selection

Does your paper address subitem 5-iv?
Copy and paste relevant sections from the manuscript (include quotes in quotation marks "like this" to indicate direct quotes from your manuscript), or elaborate on this item by providing additional information not in the ms, or briefly explain why the item is not applicable/relevant for your study

Subitem 5-iv is not applicable to the following manuscript.

5-v) Ensure replicability by publishing the source code, and/or providing screenshots/screen-capture video, and/or providing flowcharts of the algorithms used
Ensure replicability by publishing the source code, and/or providing screenshots/screen-capture video, and/or providing flowcharts of the algorithms used. Replicability (i.e., other researchers should in principle be able to replicate the study) is a hallmark of scientific reporting.

	1	2	3	4	5	
subitem not at all important	●	○	○	○	○	essential

Clear selection

Does your paper address subitem 5-v?
Copy and paste relevant sections from the manuscript (include quotes in quotation marks "like this" to indicate direct quotes from your manuscript), or elaborate on this item by providing additional information not in the ms, or briefly explain why the item is not applicable/relevant for your study

Subitem 5-v is not applicable, as there is no source code, and/or screenshots/screen capture video, and/or algorithm flowchart.

5-vi) Digital preservation
Digital preservation: Provide the URL of the application, but as the intervention is likely to change or disappear over the course of the years; also make sure the intervention is archived (Internet Archive, webcitation.org, and/or publishing the source code or screenshots/videos alongside the article). As pages behind login screens cannot be archived, consider creating demo pages which are accessible without login.

	1	2	3	4	5	
subitem not at all important	●	○	○	○	○	essential

Clear selection

Does your paper address subitem 5-vi?
Copy and paste relevant sections from the manuscript (include quotes in quotation marks "like this" to indicate direct quotes from your manuscript), or elaborate on this item by providing additional information not in the ms, or briefly explain why the item is not applicable/relevant for your study

Subitem 5-vi is not applicable. The authors will preserve a copy of the emails.

5-vii) Access
Access: Describe how participants accessed the application, in what setting/context, if they had to pay (or were paid) or not, whether they had to be a member of specific group. If known, describe how participants obtained "access to the platform and Internet" [1]. To ensure access for editors/reviewers/readers, consider to provide a "backdoor" login account or demo mode for reviewers/readers to explore the application (also important for archiving purposes, see vi).

	1	2	3	4	5	
subitem not at all important	○	○	○	○	●	essential

Clear selection

Does your paper address subitem 5-vii? *
Copy and paste relevant sections from the manuscript (include quotes in quotation marks "like this" to indicate direct quotes from your manuscript), or elaborate on this item by providing additional information not in the ms, or briefly explain why the item is not applicable/relevant for your study

It was comprised of three components: (a) a paper copy and brief verbal explanation of the Canadian PA guidelines for adults 18–64 years to establish a target for their behaviour change, (b) a Polar A300 activity monitor with a charging cable and access to the Polar Flow website [41] and companion smartphone application, and (c) six weekly behavioural support emails designed to enhance autonomous motivation for PA. Components 1 and 2 were provided to participants during their baseline meeting with MB. On the basis of evidence that self-monitoring (often conducted using technology) is correlated with PA behaviour change [42], participants received a wearable activity tracker to self-monitor the frequency and intensity of their PA behaviours towards daily or longer-term goals (e.g., walking a certain distance over time). To this end, they were instructed to wear the device on their wrist daily during waking hours for the six-week intervention period, except when swimming or bathing. MB provided instructions on how to navigate the device and assisted participants in syncing the device to their computer and/or smartphone so they could review their PA data in greater detail on either the Polar website and/or its smartphone applications. Participants maintained access to the device, Polar website and companion smartphone application for the duration of the six-week intervention and had continued access to the Polar website and companion smartphone application after returning the device to MB during the post-intervention meeting.

5-viii) Mode of delivery, features/functionalities/components of the intervention and comparator, and the theoretical framework
Describe mode of delivery, features/functionalities/components of the intervention and comparator, and the theoretical framework [6] used to design them (instructional strategy [1], behaviour change techniques, persuasive features, etc., see e.g., [7, 8] for terminology). This includes an in-depth description of the content (including where it is coming from and who developed it) [1]," whether [and how] it is tailored to individual circumstances and allows users to track their progress and receive feedback" [6]. This also includes a description of communication delivery channels and – if computer-mediated communication is a component – whether communication was synchronous or asynchronous [6]. It also includes information on presentation strategies [1], including page design principles, average amount of text on pages, presence of hyperlinks to other resources, etc. [1].

	1	2	3	4	5	
subitem not at all important	○	○	○	○	●	essential

Clear selection

Does your paper address subitem 5-viii? *
Copy and paste relevant sections from the manuscript (include quotes in quotation marks "like this" to indicate direct quotes from your manuscript), or elaborate on this item by providing additional information not in the ms, or briefly explain why the item is not applicable/relevant for your study

It was designed to be self-guided so that women could access resources at any time and in any location.
Components 1 and 2 were provided to participants during their baseline meeting with MB. On the basis of evidence that self-monitoring (often conducted using technology) is correlated with PA behaviour change [42], participants received a wearable activity tracker to self-monitor the frequency and intensity of their PA behaviours towards daily or longer-term goals (e.g., walking a certain distance over time). To this end, they were instructed to wear the device on their wrist daily during waking hours for the six-week intervention period, except when swimming or bathing. MB provided instructions on how to navigate the device and assisted participants in syncing the device to their computer and/or smartphone so they could review their PA data in greater detail on either the Polar website and/or its smartphone applications.
Component 3 featured six weekly non-tailored behaviour support emails (i.e., the same content was provided to all participants, though emails were addressed to participants and participants could tailor the content [2]). This modality was chosen as emails are inexpensive, easy to administer, can offer continuous or brief support, and are commonplace in real-world settings. The frequency of emails was set to one email per week for six weeks to strike a balance between providing sufficient encouragement without becoming bothersome. The email content, which featured textual information and worksheets that participants could download and/or print, was developed by drawing on SDT principles (i.e., (a) providing autonomy support, structure, and interpersonal involvement, (b) enhancing perceptions of autonomy, competence,
and relatedness, and (c) increasing autonomous motivation) and empirically-supported motivational and behaviour change techniques [43-45]. Accordingly, emails were written using autonomy-supportive phrasing; that is, non-controlling language to enhance participants' perceptions of autonomy (i.e., perceived control over one's actions), competence (i.e., perceived mastery of tasks and skills), and relatedness (i.e., perceived belonging and connection to others), as well as their autonomous motivation for PA [46,47]. Key motivational and behaviour change techniques (selected on the basis of previous research and their relation to SDT [48-51]) were shared with participants to: (a) provide information (e.g., basic information about the multiple health benefits of regular PA on physical and mental health and to reduce the risk of chronic disease; guidelines for PA), (b) offer feedback/advice (e.g., practical advice for starting and maintaining a regular PA schedule with daily walking suggested as an activity; suggestions for how to best form PA habits and restructure the environment to aid), (c) encourage self-monitoring by teaching ways to track PA behaviour and experiences (e.g., journal activities, reflections, monitoring with wearable activity tracker), (d) promote goal setting (e.g., support to develop personally meaningful and important goals for PA that are specific, measurable, achievable, relevant, and time bound; encouragement to form action plans and coping plans), (e) foster social support (e.g., self-identify sources of support and elicit support from such sources), (f) support self-reward (e.g., self-identify rewards to obtain upon reaching their goals for PA), and (g) enhance enjoyment (e.g., self-reflect on experiences, increase awareness of how thoughts and feelings can lead to inactivity, and thus consider enjoyable activities). Other recurring themes throughout the emails included learning from trial and error, focusing on making small changes, choosing enjoyable activities, and aligning plans with personal beliefs and values. A detailed overview of content and techniques included in the emails have been previously published [38].

5-ix) Describe use parameters
Describe use parameters (e.g., intended "doses" and optimal timing for use). Clarify what instructions or recommendations were given to the user, e.g., regarding timing, frequency, heaviness of use, if any, or was the intervention used ad libitum.

	1	2	3	4	5	
subitem not at all important	○	○	○	○	●	essential

Clear selection

Does your paper address subitem 5-ix?
Copy and paste relevant sections from the manuscript (include quotes in quotation marks "like this" to indicate direct quotes from your manuscript), or elaborate on this item by providing additional information not in the ms, or briefly explain why the item is not applicable/relevant for your study

On the basis of evidence that self-monitoring (often conducted using technology) is correlated with PA behaviour change [42], participants received a wearable activity tracker to self-monitor the frequency and intensity of their PA behaviours towards daily or longer-term goals (e.g., walking a certain distance over time). To this end, they were instructed to wear the device on their wrist daily during waking hours for the six-week intervention period, except when swimming or bathing.
Component 3 featured six weekly non-tailored behaviour support emails (i.e., the same content was provided to all participants, though emails were addressed to participants and

content was provided to all participants, though emails were addressed to participants and participants could tailor the content [2]). This modality was chosen as emails are inexpensive, easy to administer, can offer continuous or brief support, and are commonplace in real-world settings. The frequency of emails was set to one email per week for six weeks to strike a balance between providing sufficient encouragement without becoming bothersome.

5-x) Clarify the level of human involvement

Clarify the level of human involvement (care providers or health professionals, also technical assistance) in the e-intervention or as co-intervention (detail number and expertise of professionals involved, if any, as well as "type of assistance offered, the timing and frequency of the support, how it is initiated, and the medium by which the assistance is delivered". It may be necessary to distinguish between the level of human involvement required for the trial, and the level of human involvement required for a routine application outside of a RCT setting (discuss under item 21 – generalizability).

	1	2	3	4	5	
subitem not at all important	○	○	○	○	◉	essential

Clear selection

Does your paper address subitem 5-x?

Copy and paste relevant sections from the manuscript (include quotes in quotation marks "like this" to indicate direct quotes from your manuscript), or elaborate on this item by providing additional information not in the ms, or briefly explain why the item is not applicable/relevant for your study

It was designed to be self-guided so that women could access resources at any time and in any location.
Components 1 and 2 were provided to participants during their baseline meeting with MB. On the basis of evidence that self-monitoring (often conducted using technology) is correlated with PA behaviour change [42], participants received a wearable activity tracker to self-monitor the frequency and intensity of their PA behaviours towards daily or longer-term goals (e.g., walking a certain distance over time). To this end, they were instructed to wear the device on their wrist daily during waking hours for the six-week intervention period, except when swimming or bathing. MB provided instructions on how to navigate the device and assisted participants in syncing the device to their computer and/or smartphone so they could review their PA data in greater detail on either the Polar website and/or its smartphone applications.
Component 3 featured six weekly non-tailored behaviour support emails (i.e., the same content was provided to all participants, though emails were addressed to participants and participants could tailor the content [2]). This modality was chosen as emails are inexpensive, easy to administer, can offer continuous or brief support, and are commonplace in real-world settings. The frequency of emails was set to one email per week for six weeks to strike a balance between providing sufficient encouragement without becoming bothersome.

5-xi) Report any prompts/reminders used

Report any prompts/reminders used: Clarify if there were prompts (letters, emails, phone calls, SMS) to use the application, what triggered them, frequency etc. It may be necessary to distinguish between the level of prompts/reminders required for the trial, and the level of prompts/reminders for a routine application outside of a RCT setting (discuss under item 21 – generalizability).

	1	2	3	4	5	
subitem not at all important	○	○	○	○	◉	essential

Clear selection

Does your paper address subitem 5-xi? *

Copy and paste relevant sections from the manuscript (include quotes in quotation marks "like this" to indicate direct quotes from your manuscript), or elaborate on this item by providing additional information not in the ms, or briefly explain why the item is not applicable/relevant for your study

Component 3 featured six weekly non-tailored behaviour support emails (i.e., the same content was provided to all participants, though emails were addressed to participants and participants could tailor the content [2]). This modality was chosen as emails are inexpensive, easy to administer, can offer continuous or brief support, and are commonplace in real-world settings. The frequency of emails was set to one email per week for six weeks to strike a balance between providing sufficient encouragement without becoming bothersome.

5-xii) Describe any co-interventions (incl. training/support)

Describe any co-interventions (incl. training/support): Clearly state any interventions that are provided in addition to the targeted eHealth intervention, as ehealth intervention may not be designed as stand-alone intervention. This includes training sessions and support [1]. It may be necessary to distinguish between the level of training required for the trial, and the level of training for a routine application outside of a RCT setting (discuss under item 21 – generalizability.

	1	2	3	4	5	
subitem not at all important	○	○	○	○	◉	essential

Clear selection

Does your paper address subitem 5-xii? *

Copy and paste relevant sections from the manuscript (include quotes in quotation marks "like this" to indicate direct quotes from your manuscript), or elaborate on this item by providing additional information not in the ms, or briefly explain why the item is not applicable/relevant for your study

MB provided instructions on how to navigate the device and assisted participants in syncing the device to their computer and/or smartphone so they could review their PA data in greater detail on either the Polar website and/or its smartphone applications. Participants maintained access to the device, Polar website and companion smartphone application for the duration of the six-week intervention and had continued access to the Polar website and companion smartphone application after returning the device to MB during the post-intervention meeting.

6a) Completely defined pre-specified primary and secondary outcome measures, including how and when they were assessed

Does your paper address CONSORT subitem 6a? *

Copy and paste relevant sections from the manuscript (include quotes in quotation marks "like this" to indicate direct quotes from your manuscript), or elaborate on this item by providing additional information not in the ms, or briefly explain why the item is not applicable/relevant for your study

A complete description of the primary (i.e., PA behaviour) and secondary outcome measures (i.e., PA-related basic psychological needs satisfaction and motivational regulations), including how and when they were used, is available in Black and Brunet [38]. Results of analyses performed for primary and secondary outcomes are reported elsewhere [7]. Pertinent to the current study, participants self-reported sociodemographic and health information (e.g., age, marital status, ethnicity, education, work status, smoking status) at baseline within an online survey using the platform SurveyMonkey. Their height (m), body mass (kg), body fat percentage, and waist circumference (cm) were measured by MB during their in-person meeting at baseline and again at post-intervention. These data were used to describe the sample.
Post-intervention (week 7), acceptability and usage patterns data were collected online using the platform SurveyMonkey. As outlined in Table 2, three open-ended questions were used. Specifically, participants were asked what they liked and disliked about the intervention and were invited to comment on points of improvement. Participants were also asked to rate their likelihood of using the wearable activity tracker in the future and to assess its value using a 5-point Likert scale. Finally, participants were presented with five questions that asked them to indicate how often they used the wearable activity tracker and checked their data on their computer and smartphone; they were asked to select the appropriate response from a list of possible responses.

6a-i) Online questionnaires: describe if they were validated for online use and apply CHERRIES items to describe how the questionnaires were designed/deployed

If outcomes were obtained through online questionnaires, describe if they were validated for online use and apply CHERRIES items to describe how the questionnaires were designed/deployed [9].

	1	2	3	4	5	
subitem not at all important	◉	○	○	○	○	essential

Clear selection

Does your paper address subitem 6a-i?

Copy and paste relevant sections from manuscript text

The closed- and opened- ended questions were designed by the authors.

6a-ii) Describe whether and how "use" (including intensity of use/dosage) was defined/measured/monitored

Describe whether and how "use" (including intensity of use/dosage) was defined/measured/monitored (logins, logfile analysis, etc.). Use/adoption metrics are important process outcomes that should be reported in any ehealth trial.

	1	2	3	4	5	
subitem not at all important	○	○	○	◉	○	essential

Clear selection

Does your paper address subitem 6a-ii?

Copy and paste relevant sections from manuscript text

Post-intervention (week 7), acceptability and usage patterns data were collected online using the platform SurveyMonkey. As outlined in Table 2, three open-ended questions were used. Specifically, participants were asked what they liked and disliked about the intervention and were invited to comment on points of improvement. Participants were also asked to rate their likelihood of using the wearable activity tracker in the future and to assess its value using a 5-point Likert scale. Finally, participants were presented with five questions that asked them to indicate how often they used the wearable activity tracker and checked their data on their computer and smartphone; they were asked to select the appropriate response from a list of possible responses.

6a-iii) Describe whether, how, and when qualitative feedback from participants was obtained

Describe whether, how, and when qualitative feedback from participants was obtained (e.g., through emails, feedback forms, interviews, focus groups).

	1	2	3	4	5	
subitem not at all important	○	○	○	○	◉	essential

Clear selection

Does your paper address subitem 6a-iii?

Copy and paste relevant sections from manuscript text

Post-intervention (week 7), acceptability and usage patterns data were collected online using the platform SurveyMonkey. As outlined in Table 2, three open-ended questions were used. Specifically, participants were asked what they liked and disliked about the intervention and were invited to comment on points of improvement. Participants were also asked to rate their likelihood of using the wearable activity tracker in the future and to assess its value using a 5-point Likert scale. Finally, participants were presented with five questions that asked them to indicate how often they used the wearable activity tracker and checked their data on their computer and smartphone; they were asked to select the appropriate response from a list of possible responses.

6b) Any changes to trial outcomes after the trial commenced, with reasons

Does your paper address CONSORT subitem 6b? *

Copy and paste relevant sections from the manuscript (include quotes in quotation marks "like this" to indicate direct quotes from your manuscript), or elaborate on this item by providing additional information not in the ms, or briefly explain why the item is not applicable/relevant for your study

Subitem 6b is not applicable for the following study because there were no changes to trial outcomes after the trial commenced.

7a) How sample size was determined

NPT: When applicable, details of whether and how the clustering by care provides or centers was addressed

7a-i) Describe whether and how expected attrition was taken into account when calculating the sample size

Describe whether and how expected attrition was taken into account when calculating the sample size.

	1	2	3	4	5	
subitem not at all important	○	○	○	○	◉	essential

Clear selection

Does your paper address subitem 7a-i?

Copy and paste relevant sections from manuscript title (include quotes in quotation marks "like this" to indicate direct quotes from your manuscript), or elaborate on this item by providing additional information not in the ms, or briefly explain why the item is not applicable/relevant for your study

As previously outlined [38], the sample size for the trial was determined a priori based on the primary objective (i.e., to determine whether there were significant within-group changes in PA and significant between-group differences in change in PA). As the objective of the current study was to explore participants' acceptability of a theory-driven, email-delivered intervention, only those assigned to the main intervention arm were included herein.

7b) When applicable, explanation of any interim analyses and stopping guidelines

Does your paper address CONSORT subitem 7b? *

Copy and paste relevant sections from the manuscript (include quotes in quotation marks "like this" to indicate direct quotes from your manuscript), or elaborate on this item by providing additional information not in the ms, or briefly explain why the item is not applicable/relevant for your study

Subitem 7b is not applicable for the following study because no interim analyses and stopping guidelines occurred throughout the duration of the trial.

8a) Method used to generate the random allocation sequence

NPT: When applicable, how care providers were allocated to each trial group

Does your paper address CONSORT subitem 8a? *

Copy and paste relevant sections from the manuscript (include quotes in quotation marks "like this" to indicate direct quotes from your manuscript), or elaborate on this item by providing additional information not in the ms, or briefly explain why the item is not applicable/relevant for your study

Participants were allocated using permuted blocks of three and six via a web-based randomization software program (Sealed Envelope Ltd., 2017).

8b) Type of randomisation; details of any restriction (such as blocking and block size)

Does your paper address CONSORT subitem 8b? *

Copy and paste relevant sections from the manuscript (include quotes in quotation marks "like this" to indicate direct quotes from your manuscript), or elaborate on this item by providing additional information not in the ms, or briefly explain why the item is not applicable/relevant for your study

Participants were allocated using permuted blocks of three and six via a web-based randomization software program (Sealed Envelope Ltd., 2017). Randomization was carried out by an independent researcher who was not involved in the trial, and arm allocation was subsequently conveyed to participants by MB via email.

9) Mechanism used to implement the random allocation sequence (such as sequentially numbered containers), describing any steps taken to conceal the sequence until interventions were assigned

Does your paper address CONSORT subitem 9? *

Copy and paste relevant sections from the manuscript (include quotes in quotation marks "like this" to indicate direct quotes from your manuscript), or elaborate on this item by providing additional information not in the ms, or briefly explain why the item is not applicable/relevant for your study

Randomization was carried out by an independent researcher who was not involved in the trial, and arm allocation was subsequently conveyed to participants by MB via email.

10) Who generated the random allocation sequence, who enrolled participants, and who assigned participants to interventions

Does your paper address CONSORT subitem 10? *

Copy and paste relevant sections from the manuscript (include quotes in quotation marks "like this" to indicate direct quotes from your manuscript), or elaborate on this item by providing additional information not in the ms, or briefly explain why the item is not applicable/relevant for your study

MB first provided study information and assessed interested persons for eligibility. Randomization was carried out by an independent researcher who was not involved n the trial, and arm allocation was subsequently conveyed to participants by MB via email.

11a) If done, who was blinded after assignment to interventions (for example, participants, care providers, those assessing outcomes) and how

NPT: Whether or not administering co-interventions were blinded to group assignment

11a-i) Specify who was blinded, and who wasn't

Specify who was blinded, and who wasn't. Usually, in web-based trials it is not possible to blind the participants [1, 3] (this should be clearly acknowledged), but it may be possible to blind outcome assessors, those doing data analysis or those administering co-interventions (if any).

	1	2	3	4	5	
subitem not at all important	○	○	○	○	◉	essential

Clear selection

Does your paper address subitem 11a-i? *

Copy and paste relevant sections from the manuscript (include quotes in quotation marks "like this" to indicate direct quotes from your manuscript), or elaborate on this item by providing additional information not in the ms, or briefly explain why the item is not applicable/relevant for your study

Due to the nature of the arms and the researchers' active role in delivering the intervention, respectively, participants and the researchers were aware of the allocated arm (i.e., unblinded); neither were blinded after assignment to arms.

11a-ii) Discuss e.g., whether participants knew which intervention was the "intervention of interest" and which one was the "comparator"

Informed consent procedures (4a-ii) can create biases and certain expectations - discuss e.g., whether participants knew which intervention was the "intervention of interest" and which one was the "comparator".

	1	2	3	4	5	
subitem not at all important	○	○	○	○	◉	essential

Clear selection

Does your paper address subitem 11a-ii?

Copy and paste relevant sections from the manuscript (include quotes in quotation marks "like this" to indicate direct quotes from your manuscript), or elaborate on this item by providing additional information not in the ms, or briefly explain why the item is not applicable/relevant for your study

Due to the nature of the arms and the researchers' active role in delivering the intervention, respectively, participants and the researchers were aware of the allocated arm (i.e., unblinded); neither were blinded after assignment to arms.

11b) If relevant, description of the similarity of interventions

(this item is usually not relevant for ehealth trials as it refers to similarity of a placebo or sham intervention to a active medication/intervention)

Does your paper address CONSORT subitem 11b? *

Copy and paste relevant sections from the manuscript (include quotes in quotation marks "like this" to indicate direct quotes from your manuscript), or elaborate on this item by providing additional information not in the ms, or briefly explain why the item is not applicable/relevant for your study

Table 1 provides an overview of the three arms, showcasing their similarities.

12a) Statistical methods used to compare groups for primary and secondary outcomes

NPT: When applicable, details of whether and how the clustering by care providers or centers was addressed

Does your paper address CONSORT subitem 12a? *

Copy and paste relevant sections from the manuscript (include quotes in quotation marks "like this" to indicate direct quotes from your manuscript), or elaborate on this item by providing additional information not in the ms, or briefly explain why the item is not applicable/relevant for your study

Subitem 12a is not applicable. Results of analyses performed for primary and secondary outcomes are reported elsewhere [7].

12a-i) Imputation techniques to deal with attrition / missing values

Imputation techniques to deal with attrition / missing values: Not all participants will use the intervention/comparator as intended and attrition is typically high in ehealth trials. Specify how participants who did not use the application or dropped out from the trial were treated in the statistical analysis (a complete case analysis is strongly discouraged, and simple imputation techniques such as LOCF may also be problematic [4]).

	1	2	3	4	5	
subitem not at all important	○	◉	○	○	○	essential

Clear selection

Does your paper address subitem 12a-i? *

Copy and paste relevant sections from the manuscript (include quotes in quotation marks "like this" to indicate direct quotes from your manuscript), or elaborate on this item by providing additional information not in the ms, or briefly explain why the item is not applicable/relevant for your study

Subitem 12a-i is not applicable. Imputation techniques were not used to deal with attrition/missing values.

12b) Methods for additional analyses, such as subgroup analyses and adjusted analyses

Does your paper address CONSORT subitem 12b? *

Copy and paste relevant sections from the manuscript (include quotes in quotation marks "like this" to indicate direct quotes from your manuscript), or elaborate on this item by providing additional information not in the ms, or briefly explain why the item is not applicable/relevant for your study

Descriptive statistics were used to summarize the sample characteristics and responses to the acceptability questions with fixed-response options; Statistical Package for Social Sciences (SPSS) (version 26; IBM Corporation, Armonk, NY, USA) was used to conduct these analyses. Content analysis using a deductive approach [52] was carried out by two graduate students familiar with the trial but not otherwise involved in its development or execution (SS and JP) to generate themes derived from participants' free-text responses to the open-ended acceptability questions. First, JP and SS read through all the textual data to obtain a general understanding. Next, they created a preliminary generation of codes based on the acceptability questions and searched for themes independently. Discrepancies were then discussed, and JB became involved; she reviewed each theme and all open-ended responses to the survey, and discussions took place until a consensus was reached. Microsoft Word was used to store, code, and analyses open-ended responses to the survey.

X26) REB/IRB Approval and Ethical Considerations [recommended as subheading under "Methods"] (not a CONSORT item)

X26-i) Comment on ethics committee approval

	1	2	3	4	5	
subitem not at all important	○	○	○	○	◉	essential

Clear selection

Does your paper address subitem X26-i?

Copy and paste relevant sections from the manuscript (include quotes in quotation marks "like this" to indicate direct quotes from your manuscript), or elaborate on this item by providing additional information not in the ms, or briefly explain why the item is not applicable/relevant for your study

The trial protocol was approved by the Institutional Review Board at the University of Ottawa (H-06-18-437) before recruitment and data collection began. Participation was voluntary. All participants gave informed consent digitally in agreement with the Declaration of Helsinki, and were able to withdraw from the study at any time. Participants received no financial compensations for taking part in this study.

x26-ii) Outline informed consent procedures

Outline informed consent procedures e.g., if consent was obtained offline or online (how? Checkbox, etc.?), and what information was provided (see 4a-ii). See [6] for some items to be included in informed consent documents.

	1	2	3	4	5	
subitem not at all important	○	○	○	○	◉	essential

Clear selection

Does your paper address subitem X26-ii?

Copy and paste relevant sections from the manuscript (include quotes in quotation marks "like this" to indicate direct quotes from your manuscript), or elaborate on this item by providing additional information not in the ms, or briefly explain why the item is not applicable/relevant for your study

MB first provided study information and assessed interested persons for eligibility. Once eligibility criteria were confirmed, persons were sent an email that included a link to access an online survey platform (i.e., SurveyMonkey). Upon accessing the platform, persons were required to read a consent form and digitally sign it, after which they were redirected to complete self-reported measures.

X26-iii) Safety and security procedures

Safety and security procedures, incl. privacy considerations, and any steps taken to reduce the likelihood or detection of harm (e.g., education and training, availability of a hotline)

	1	2	3	4	5	
subitem not at all important	○	○	○	○	◉	essential

Clear selection

Does your paper address subitem X26-iii?

Copy and paste relevant sections from the manuscript (include quotes in quotation marks "like this" to indicate direct quotes from your manuscript), or elaborate on this item by providing additional information not in the ms, or briefly explain why the item is not applicable/relevant for your study

All data were de-identified prior to analysis. Adverse events and unanticipated effects during the duration of the trial were collected via self-report; none were reported.

RESULTS

13a) For each group, the numbers of participants who were randomly assigned, received intended treatment, and were analysed for the primary outcome

NPT: The number of care providers or centers performing the intervention in each group and the number of patients treated by each care provider in each center

Does your paper address CONSORT subitem 13a? *

Copy and paste relevant sections from the manuscript (include quotes in quotation marks "like this" to indicate direct quotes from your manuscript), or elaborate on this item by providing additional information not in the ms, or briefly explain why the item is not applicable/relevant for your study

Recruitment started in September 2018 and ended in March 2019 when the target sample size was reached, and follow-up assessments took place until August 2019; a flow diagram including the number of participants who were randomly assigned, who received the intervention, and who were analyzed is presented elsewhere [7], as are the reasons for exclusions and losses. Fifteen participants were randomized to the main intervention arm; of these, 14 (93.3%) provided acceptability data post-intervention (see Table 3 for characteristics; characteristics for all participants are presented elsewhere [7]). Due to efforts to ensure anonymity and data protection, it was not possible to ascertain the reason(s) for dropout). The analyzed sample of women were between 18-63 years of age and most (74.3%, n=11/15) self-identified as White. There were no reports of adverse events or unanticipated effects during the duration of the trial.

13b) For each group, losses and exclusions after randomisation, together with reasons

Does your paper address CONSORT subitem 13b? (NOTE: Preferably, this is shown in a CONSORT flow diagram) *

Copy and paste relevant sections from the manuscript (include quotes in quotation marks "like this" to indicate direct quotes from your manuscript), or elaborate on this item by providing additional information not in the ms, or briefly explain why the item is not applicable/relevant for your study

Recruitment started in September 2018 and ended in March 2019 when the target sample size was reached, and follow-up assessments took place until August 2019; a flow diagram including the number of participants who were randomly assigned, who received the intervention, and who were analyzed is presented elsewhere [7], as are the reasons for exclusions and losses.

13b-i) Attrition diagram

Strongly recommended: An attrition diagram (e.g., proportion of participants still logging in or using the intervention/comparator in each group plotted over time, similar to a survival curve) or other figures or tables demonstrating usage/dose/engagement.

	1	2	3	4	5	
subitem not at all important	◉	○	○	○	○	essential

Clear selection

Does your paper address subitem 13b-i?

Copy and paste relevant sections from the manuscript or cite the figure number if applicable (include quotes in quotation marks "like this" to indicate direct quotes from

your manuscript), or elaborate on this item by providing additional information not in the ms, or briefly explain why the item is not applicable/relevant for your study

Subitem 13b-i is not applicable. Given the nature of the intervention, it was not possible to collect these data.

14a) Dates defining the periods of recruitment and follow-up

Does your paper address CONSORT subitem 14a? *
Copy and paste relevant sections from the manuscript (include quotes in quotation marks "like this" to indicate direct quotes from your manuscript), or elaborate on this item by providing additional information not in the ms, or briefly explain why the item is not applicable/relevant for your study

Recruitment started in September 2018 and ended in March 2019 when the target sample size was reached, and follow-up assessments took place until August 2019.

14a-i) Indicate if critical "secular events" fell into the study period
Indicate if critical "secular events" fell into the study period, e.g., significant changes in Internet resources available or "changes in computer hardware or Internet delivery resources"

	1	2	3	4	5	
subitem not at all important	◉	○	○	○	○	essential

Clear selection

Does your paper address subitem 14a-i?
Copy and paste relevant sections from the manuscript (include quotes in quotation marks "like this" to indicate direct quotes from your manuscript), or elaborate on this item by providing additional information not in the ms, or briefly explain why the item is not applicable/relevant for your study

Subitem 14a-i is not applicable for the following study because no critical "secular events" fell into the study period.

14b) Why the trial ended or was stopped (early)

Does your paper address CONSORT subitem 14b? *
Copy and paste relevant sections from the manuscript (include quotes in quotation marks "like this" to indicate direct quotes from your manuscript), or elaborate on this item by providing additional information not in the ms, or briefly explain why the item is not applicable/relevant for your study

Subitem 14b is not applicable for the following study. The trial ended as planned. Specifically, recruitment started in September 2018 and ended in March 2019 when the target sample size was reached.

15) A table showing baseline demographic and clinical characteristics for each group
NPT: When applicable, a description of care providers (case volume, qualification, expertise, etc.) and centers (volume) in each group

Does your paper address CONSORT subitem 15? *
Copy and paste relevant sections from the manuscript (include quotes in quotation marks "like this" to indicate direct quotes from your manuscript), or elaborate on this item by providing additional information not in the ms, or briefly explain why the item is not applicable/relevant for your study

Fifteen participants were randomized to the main intervention arm; of these, 14 (93.3%) provided acceptability data post-intervention (see Table 3 for characteristics; characteristics for all participants are presented elsewhere [7]). Due to efforts to ensure anonymity and data protection, it was not possible to ascertain the reason(s) for dropout). The analyzed sample of women were between 18-63 years of age and most (74.3%, n=11/15) self-identified as White. There were no reports of adverse events or unanticipated effects during the duration of the trial.

15-i) Report demographics associated with digital divide issues
In ehealth trials it is particularly important to report demographics associated with digital divide issues, such as age, education, gender, social-economic status, computer/Internet/ehealth literacy of the participants, if known.

	1	2	3	4	5	
subitem not at all important	○	○	○	○	◉	essential

Clear selection

Does your paper address subitem 15-i? *
Copy and paste relevant sections from the manuscript (include quotes in quotation marks "like this" to indicate direct quotes from your manuscript), or elaborate on this item by providing additional information not in the ms, or briefly explain why the item is not applicable/relevant for your study

Please refer to Table 3. Sociodemographic and health profile of participants at baseline who provided acceptability data post-intervention (n=14) to address subitem 15-i.

16) For each group, number of participants (denominator) included in each analysis and whether the analysis was by original assigned groups

16-i) Report multiple "denominators" and provide definitions
Report multiple "denominators" and provide definitions: Report N's (and effect sizes) "across a range of study participation [and use] thresholds" [1], e.g., N exposed, N consented, N used more than x times, N used more than y weeks, N participants "used" the intervention/comparator at specific pre-defined time points of interest (in absolute and relative numbers per group). Always clearly define "use" of the intervention.

	1	2	3	4	5	
subitem not at all important	○	○	○	○	◉	essential

Clear selection

Does your paper address subitem 16-i? *
Copy and paste relevant sections from the manuscript (include quotes in quotation marks "like this" to indicate direct quotes from your manuscript), or elaborate on this item by providing additional information not in the ms, or briefly explain why the item is not applicable/relevant for your study

The denominator is 14 for quantitative analyses.

16-ii) Primary analysis should be intent-to-treat
Primary analysis should be intent-to-treat, secondary analyses could include comparing only "users", with the appropriate caveats that this is no longer a randomized sample (see 18-i).

	1	2	3	4	5	
subitem not at all important	◉	○	○	○	○	essential

Clear selection

Does your paper address subitem 16-ii?
Copy and paste relevant sections from the manuscript (include quotes in quotation marks "like this" to indicate direct quotes from your manuscript), or elaborate on this item by providing additional information not in the ms, or briefly explain why the item is not applicable/relevant for your study

Subitem 16-ii is not applicable. No inferential statistical analyses were conducted.

17a) For each primary and secondary outcome, results for each group, and the estimated effect size and its precision (such as 95% confidence interval)

Does your paper address CONSORT subitem 17a? *
Copy and paste relevant sections from the manuscript (include quotes in quotation marks "like this" to indicate direct quotes from your manuscript), or elaborate on this item by providing additional information not in the ms, or briefly explain why the item is not applicable/relevant for your study

Recruitment started in September 2018 and ended in March 2019 when the target sample size was reached, and follow-up assessments took place until August 2019; a flow diagram including the number of participants who were randomly assigned, who received the intervention, and who were analyzed is presented elsewhere [7], as are the reasons for exclusions and losses.

17a-i) Presentation of process outcomes such as metrics of use and intensity of use
In addition to primary/secondary (clinical) outcomes, the presentation of process outcomes such as metrics of use and intensity of use (dose, exposure) and their operational definitions is critical. This does not only refer to metrics of attrition (13-b) (often a binary variable), but also to more continuous exposure metrics such as "average session length". These must be accompanied by a technical description how a metric like a "session" is defined (e.g., timeout after idle time) [1] (report under item 6a).

	1	2	3	4	5	
subitem not at all important	○	○	○	○	◉	essential

Clear selection

Does your paper address subitem 17a-i?
Copy and paste relevant sections from the manuscript (include quotes in quotation marks "like this" to indicate direct quotes from your manuscript), or elaborate on this item by providing additional information not in the ms, or briefly explain why the item is not applicable/relevant for your study

Subitem 17a is not applicable. This manuscript reports analyses for a single group.

17b) For binary outcomes, presentation of both absolute and relative effect sizes is recommended

Does your paper address CONSORT subitem 17b? *
Copy and paste relevant sections from the manuscript (include quotes in quotation marks "like this" to indicate direct quotes from your manuscript), or elaborate on this item by providing additional information not in the ms, or briefly explain why the item is not applicable/relevant for your study

Item 17b is not applicable for the following study because no inferential statistical analyses were conducted.

18) Results of any other analyses performed, including subgroup analyses and adjusted analyses, distinguishing pre-specified from exploratory

Does your paper address CONSORT subitem 18? *
Copy and paste relevant sections from the manuscript (include quotes in quotation marks "like this" to indicate direct quotes from your manuscript), or elaborate on this item by providing additional information not in the ms, or briefly explain why the item is not applicable/relevant for your study

Subitem 18 is not applicable because no additional subgroup analyses and adjusted analyses were performed.

18-i) Subgroup analysis of comparing only users
A subgroup analysis of comparing only users is not uncommon in ehealth trials, but if done, it must be stressed that this is a self-selected sample and no longer an unbiased sample from a randomized trial (see 16-iii).

	1	2	3	4	5	
subitem not at all important	○	○	○	◉	○	essential

Clear selection

Does your paper address subitem 18-i?
Copy and paste relevant sections from the manuscript (include quotes in quotation marks "like this" to indicate direct quotes from your manuscript), or elaborate on this item by providing additional information not in the ms, or briefly explain why the item is not applicable/relevant for your study

The manuscript reports results from those receiving the main intervention as data pertaining to the intervention were collected (and was thus not relevant to other groups).

19) All important harms or unintended effects in each group
(for specific guidance see CONSORT for harms)

Does your paper address CONSORT subitem 19? *
Copy and paste relevant sections from the manuscript (include quotes in quotation marks "like this" to indicate direct quotes from your manuscript), or elaborate on this item by providing additional information not in the ms, or briefly explain why the item is not applicable/relevant for your study

Subitem 19 is not applicable because there were no reports of adverse events or unanticipated effects during the duration of the trial.

19-i) Include privacy breaches, technical problems
Include privacy breaches, technical problems. This does not only include physical "harm" to participants, but also incidents such as perceived or real privacy breaches [1], technical problems, and other unexpected/unintended incidents. "Unintended effects" also includes unintended positive effects [2].

	1	2	3	4	5	
subitem not at all important	◉	○	○	○	○	essential

Clear selection

Does your paper address subitem 19-i?
Copy and paste relevant sections from the manuscript (include quotes in quotation marks "like this" to indicate direct quotes from your manuscript), or elaborate on this item by providing additional information not in the ms, or briefly explain why the item is not applicable/relevant for your study

Subitem 19-i is not applicable because no privacy breaches or technical problems occurred.

19-ii) Include qualitative feedback from participants or observations from staff/researchers
Include qualitative feedback from participants or observations from staff/researchers, if available, on strengths and shortcomings of the application, especially if they point to unintended/unexpected effects or uses. This includes (if available) reasons for why people did or did not use the application as intended by the developers.

	1	2	3	4	5	
subitem not at all important	○	○	○	○	◉	essential

Clear selection

Does your paper address subitem 19-ii?
Copy and paste relevant sections from the manuscript (include quotes in quotation marks "like this" to indicate direct quotes from your manuscript), or elaborate on this item by providing additional information not in the ms, or briefly explain why the item is not applicable/relevant for your study

The 14 women who completed the post-intervention survey provided a total of 41 responses to three open-ended acceptability questions (see Table 2). The final themes comprise their expressed likes and dislikes pertaining to the intervention, as well as recommendations for future implementation; these are presented below, along with quotations from free-text responses to illustrate key ideas. To protect confidentiality, participants were assigned pseudonyms, which are included next to each quotation.
Participants' responses suggested they were mostly enthusiastic about the email-delivered intervention. Overall, the intervention was considered useful because it helped keep them 'on track and accountable' (Anna) and offered a sense of accountability in a non-pressuring manner. Participants expressed positive regard for the emails and felt they were an important element of the intervention. Specifically, they mentioned that the emails provided useful and helpful informational support, offered new ideas and activities they could try to meet their PA goals, and helped them learn how to develop feasible and/or alternative PA goals. Moreover, participants appreciated that the emails contained relevant information as they were 'based around health for women' (Jackie). They also relayed that the information delivered within the emails helped increase their awareness of their PA behaviour by prompting self-reflection; as Michelle highlighted, 'It got me to think about my actions and where there were problems.' Beyond the content, participants said the frequency of emails (i.e., once per week) was appropriate; weekly emails helped keep them engaged and served as 'good reminders to reflect on making change' (Peyton). Further, the design and layout of the activity sheets embedded within the emails were appreciated. Last, participants' responses demonstrated that they positively perceived the wearable activity tracker as it offered them an objective measure of their PA; these data were meaningful because it increased participants' awareness of their current PA levels and helped identify discrepancies between their current and desired levels. The device also served as a visual reminder to participants that their PA levels were being tracked, and thus encouraged them to be more active.
Although participants largely appreciated the intervention, they also reported dislikes that may have interfered with the acceptability of the invention and limited their motivation for participation. Their dislikes were related to the style of messaging within the emails (i.e., non-tailored content), design and delivery of the emails (i.e., textual presentation, spacing, timing, automation), intervention delivery schedule, and device factors. In terms of the style of messaging, participants said that receiving non-tailored content was suboptimal because the information provided was not specific to their individual PA needs and preferences; this, in turn, reduced their perceived value and utility. A key issue undermining the potential value and utility of the emails was that they only offered 'passive guidance' (Maya), and consequently did not serve to increase participants' willingness and capability to successfully engage in PA in their daily lives.
In terms of the design and delivery of the emails, there were three specific issues. First, while they could be informative, emails were automated and therefore did not allow for two-way communication between participants and MB. Participants cited two-way communication as important for ensuring they engaged with the intervention and felt that having to submit their completed activity sheets would have increased their motivation to complete them. Indeed, the lack of accountability due to the one-way emailing design did little to enhance Priya's motivation to change her PA behaviour as it was 'hard only being accountable to myself.' Relatedly, participants disliked that the one-way emailing did not afford them opportunities to share difficulties/challenges/failures with MB and receive tailored messaging in return; their responses suggested that this would have been beneficial to prevent negative feelings about not achieving their PA goals. Second, participants felt the emails lacked examples to visually help them produce their own PA programs, which left them feeling overwhelmed and unsure how to proceed. Maya explained that 'choosing the best activity to make the most noticeable improvement is difficult as there are so many choices of forms of exercise' and expressed disappointment that the emails 'did not lay out example exercise schedules to follow.' Third, some expressed dissatisfaction related to the design (i.e., length and density) and delivery (i.e., spacing and timing) of the emails. For instance, Leah and Sam described the emails as 'long' and 'text-heavy,' respectively, whereas Julie explained that she 'had trouble completing the weekly self-assessments' because there was not enough time to do so in-between emails. The time of the week that emails were sent was also raised as an issue; Jackie suggested that she would have been more inclined to read them had they arrived towards the end of week, as opposed to mid-week. Moreover, the rolling-recruitment strategy adopted in this study presented challenges for some recruited during Winter months. Kristen gave specific reasons for difficulty engaging in PA (e.g., below-freezing temperatures, icy sidewalks, winter blues), and noted that the timing of the intervention was suboptimal because she had 'a really hard time starting new active things outside in the winter.' Finally, participants expressed dissatisfaction with both the physical appearance and accuracy of the Polar A300 activity monitor. That is, they found the monitor to be 'cumbersome' and 'aesthetically unappealing,' and they worried that it did not register some of their activities, leading to an underestimation of their PA levels.
The open-ended responses to the survey questions were also valuable in understanding what may help enhance participants' acceptability of future iterations of the intervention, and thus, perhaps their motivation, uptake, engagement, and adherence. While some indicated there was nothing they would change about the intervention, others made recommendations relating to its specific components (i.e., emails, device). As a means of improving the emails, participants suggested they be shorter, less text-heavy, and embedded activity sheets be revised to contain more background information. They also suggested emails be personalized to provide tailored content for each individual and be delivered when desired by participants. Further, participants suggested adding multimedia components to the emails (e.g., video/audio content, graphical illustrations), including 'incorporate some videos' (Anna) and 'provide exercise schedules to use either at home or at a gym' (Peyton). Moreover, in reference to the wearable activity tracker, participants suggested that it be swapped for a device that is more aesthetic and capable of accurately monitoring PA by including a built-in heart rate monitor.
Other recommendations based on participants' responses related to the overall intervention approach/methods. These included: (a) offering a booklet (either digital or print) that collates all emails and supporting materials to increase accessibility, and (b) requiring greater participant involvement by requesting that activity sheets be returned for review. Indeed, one participant indicated that 'reviewing each week's assignment with a person who could provide advice/support would help.' As well, participants desired two-way communication and suggested the intervention provide both automated (prescheduled) emails and opportunities to interact with someone to receive further support as needed. For the latter, proposed adaptations to the intervention included introducing 'a meeting halfway' (Maya), back-and-forth emailing, and/or phone calls. Others suggested combining the email-delivered intervention with 'group or one-on-one fitness classes/walks' (Priya), although participants did not offer opinions on desired context of such sessions (e.g., who should lead them, where they should occur, how frequently they should be offered).

DISCUSSION

22) Interpretation consistent with results, balancing benefits and harms, and considering other relevant evidence
NPT: In addition, take into account the choice of the comparator, lack of or partial blinding, and unequal expertise of care providers or centers in each group

22-i) Restate study questions and summarize the answers suggested by the data, starting with primary outcomes and process outcomes (use)
Restate study questions and summarize the answers suggested by the data, starting with primary outcomes and process outcomes (use).

	1	2	3	4	5	
subitem not at all important	○	○	○	○	◉	essential

Clear selection

Does your paper address subitem 22-i? *
Copy and paste relevant sections from the manuscript (include quotes in quotation marks "like this" to indicate direct quotes from your manuscript), or elaborate on this item by providing additional information not in the ms, or briefly explain why the item is not applicable/relevant for your study

The quantitative data suggested good overall acceptability and usability for the wearable activity tracker as it was rated as being valuable and likely to be used by most. Correspondingly, participants' responses to open-ended questions suggested they valued the objective PA data the tracker provided because it helped them better understand their current PA levels (or lack thereof) and encouraged them to make changes. However, certain design factors (e.g., size, lack of aesthetic appeal) did affect the acceptability of the device and participants' motivation to wear it. The quantitative data on the usage patterns of the Polar website and companion smartphone application showed variability amongst participants; some used these tools more frequently, whereas others used them a limited number of times or not at all. The latter is surprising because the emails included statements that could have prompted more frequent use of the Polar website and companion smartphone application (e.g., they encouraged self-monitoring and proposed the website and application as means to do so). Potential reasons for the variation in participant engagement with these technology-based tools were not queried, but based on prior research [53], they may include: (a) differing preferences for how to monitor PA (e.g., a preference for printed PA logs), (b) limited opportunities to interact with the tools, (c) feelings of disappointment triggered by results (e.g., failing to reach step count goal), (d) low perceived utility, (e) increased perceived burden from manual tracking, (f) lack of understanding or trust in the data, making it seem pointless to review its data, and (g) concerns about privacy. As well, perceived usability or ease of use could have influenced usage patterns [54]. That is, previous experience (or lack thereof) using digital applications in general, design flaws, and/or technical issues could also account for variability in participants' engagement with the device itself and with the Polar website and companion smartphone application. Unfortunately, there was no interviewer to intervene and ask follow-up questions (unlike in interviews), which could have given insight into the reasons and offer suggestions to increase usage. To fill this knowledge gap, synchronous, in-depth interviewing (by telephone, online, or face-to-face) could be used to delve deeper into the reasons. Regardless of the reason(s) behind (non)usage patterns, it would be helpful to address low device and applications usage issues in future iterations of this intervention as it is reasonable to expect it may undermine effectiveness.
Participants' responses to the open-ended questions suggest that an email-based intervention has potential to be well-received by women who are overweight or obese and insufficiently active because of what it can offer. Specifically, women in this study felt the emails: (a) provided useful and helpful informational support, (b) offered new ideas/strategies to meet PA goals, (c) taught them how to develop feasible and/or alternative PA goals, and most importantly, (d) prompted self-reflection on their PA behaviour, thus motivating them make the changes necessary to progress towards their personal PA goals. Although there is not enough evidence to draw conclusions about the effectiveness of this approach, findings provide evidence that email-delivered interventions should be considered to address barriers associated with traditional, face-to-face health-related interventions (i.e., by reducing costs [e.g., monetary, time] and increasing convenience and accessibility [e.g., for isolated and stigmatized groups] [55]. Nevertheless, participants' responses also shed light on potential threats to acceptability and point towards what could be improved. Primarily, participants raised concerns about the content, format, and style/design of the emails, as well as the predetermined, researcher-set delivery schedule. This suggests emails should be tailored and personalized to the individual (both in terms of their content and delivery protocol [i.e., timing, spacing]), and that some text should be replaced or emphasized with multimedia (e.g., videos, graphics) to foster better content engagement. Considering the extent of the suggested revision, it will be important to conduct a sufficiently powered trial to test the effects of the revised emails (and delivery protocol) on PA behaviour.
Finally, issues were raised concerning the fully-automated nature of the intervention. Although participants were provided with a description of the intervention, their responses suggest that rather than being passive recipients of information via one-way emailing, they would have preferred the opportunity to actively participate through interactions with MB (e.g., via two-way emailing, phone calls, meetings) or with other women (e.g., via PA classes). This finding is not surprising given women's desires for support from credible health experts [56] and engaging with other participants during PA interventions [57]. With these desires in mind, developing tailored interventions that allow women's needs, wants, and preferences to dictate the level of human involvement (i.e., the type of support offered, the timing and frequency of the support, how it is initiated, and the medium by which the support is delivered) may be necessary to promote experiential cognitive and emotional responses that facilitate PA during and after the intervention. Accordingly, a stepped care model [58] may be a solution; that is, a fully-automated intervention could be initiated first, followed by increased human involvement as needed (e.g., stepping up the level of provider involvement to more intensive coaching or other forms of support as needed). Future work is needed to determine if the introduction of this model renders this intervention more effective, and to explore the potential ramifications of featuring additional components (e.g., costs) and ensure it could be implemented outside of a research setting. Arguably, fully-automated interventions are beneficial as they can be initiated by administrative personnel or through pre-programmed email-based software, and thus typically incur fewer costs [55,59,60]. In contrast, increasing provider involvement or carrying out PA classes may have higher implementation costs as they may require more time to educate providers and participants, facilitate engagement, and support users. Thus, the decision to increase support, while perhaps more acceptable and possibly more impactful, should be considered in light of available resources. These are important issues to explore in future studies via cost metrics to gauge potential sustainability.

22-ii) Highlight unanswered new questions, suggest future research
Highlight unanswered new questions, suggest future research.

	1	2	3	4	5	
subitem not at all important	○	○	○	○	◉	essential

Clear selection

Does your paper address subitem 22-ii?
Copy and paste relevant sections from the manuscript (include quotes in quotation marks "like this" to indicate direct quotes from your manuscript), or elaborate on this item by providing additional information not in the ms, or briefly explain why the item is not applicable/relevant for your study

Developing tailored interventions that allow women's needs, wants, and preferences to dictate the level of human involvement (i.e., the type of support offered, the timing and frequency of the support, how it is initiated, and the medium by which the support is delivered) may be necessary to promote experiential cognitive and emotional responses that facilitate PA during and after the intervention. Accordingly, a stepped care model [58] may be a solution; that is, a fully-automated intervention could be initiated first, followed by increased human involvement as needed (e.g., stepping up the level of provider involvement to more intensive coaching or other forms of support as needed). Future work is needed to determine if the introduction of this model renders this intervention more effective, and to explore the potential ramifications of featuring additional components (e.g., costs) and ensure it could be implemented outside of a research setting. Arguably, fully-automated interventions are beneficial as they can be initiated by administrative personnel or through pre-programmed email-based software, and thus typically incur fewer costs [55,59,60]. In contrast, increasing provider involvement or carrying out PA classes may have higher implementation costs as they may require more time to educate providers and participants, facilitate engagement, and support users. Thus, the decision to increase support, while perhaps more acceptable and possibly more impactful, should be considered in light of available resources. These are important issues to explore in future studies via cost metrics to gauge potential sustainability.

20) Trial limitations, addressing sources of potential bias, imprecision, and, if relevant, multiplicity of analyses

20-i) Typical limitations in ehealth trials
Typical limitations in ehealth trials: Participants in ehealth trials are rarely blinded. Ehealth trials often look at a multiplicity of outcomes, increasing risk for a Type I error. Discuss biases due to non-use of the intervention/usability issues, biases through informed consent procedures, unexpected events.

	1	2	3	4	5	
subitem not at all important	○	○	○	○	◉	essential

Clear selection

Does your paper address subitem 20-i? *
Copy and paste relevant sections from the manuscript (include quotes in quotation marks "like this" to indicate direct quotes from your manuscript), or elaborate on this item by providing additional information not in the ms, or briefly explain why the item is not applicable/relevant for your study

A critical, yet unresolved issue in this study is how well participants felt the intervention and its components helped foster their PA-related psychological needs satisfaction and autonomous motivation. The latter also warrants further investigation as it serves as one indicator of implementation success. Arguably, enhancing participants' psychological needs satisfaction and autonomous motivation for PA is a necessary precondition for attaining subsequent desired change in PA behaviour. Adopting qualitative methods to conduct an in-depth investigation into how the intervention promoted or thwarted process outcomes will help to advance knowledge on how these key constructs can be fostered in future iterations of this intervention.
This study has limitations that could be address in future work. First, convenience sampling was used and participants were self-selected, which may have resulted in selection bias. Specifically, participants may have been more interested in changing their PA behaviour (and thus more motivated to fulfill intervention requirements) and/or more competent with technology than women in the general population; they were also well-educated. These factors could have influenced their responses and limit the applicability of the results. Second, no procedures were put in place to recruit a heterogenous sample based on sociodemographic characteristics, limiting the generalizability of the results. Future studies could use a purposive sampling method to recruit women with specific sociodemographic characteristics. Third, the exploratory nature of this study and sample characteristics did not allow for investigation of sociodemographic factors that may influence how the intervention would be received and acted upon. Fourth, important/relevant evidence regarding acceptability may not have been fully captured as usability and acceptability data were collected retrospectively using a brief questionnaire, which limited probing and follow-up question to gain more in-depth exploration of themes. Acceptability is dynamic and can be assessed prospectively, concurrently, and/or retrospectively [65]. As well, it is a multifaceted concept that includes the following constructs: affective attitude (i.e., how an individual feels about the intervention), burden (i.e., perceived amount of effort that is required to participate in the intervention), ethical (i.e., goodness of fit between intervention and individual values), coherence (i.e., participant understanding of intervention and how it works), opportunity costs (i.e., extent to which benefits, profits, or values must be given up to engage in the intervention), perceived effectiveness (i.e., extent to which intervention is perceived as likely to achieve its purpose), and self-efficacy (i.e., confidence to perform required behaviours) [65]. Depending on when acceptability is assessed and what constructs are assessed, evidence of acceptability may be different. In future studies, acceptability evaluations necessitate careful assessment of these different constructs over time. The theoretical framework of acceptability [65] may be appropriate to inform such assessments. Finally, device usability patterns were assessed via self-report, which can be prone to response bias, and no verification of whether participants read the emails and completed the activity sheets that were sent to them was carried out.

21) Generalisability (external validity, applicability) of the trial findings
NPT: External validity of the trial findings according to the intervention, comparators, patients, and care providers or centers involved in the trial

21-i) Generalizability to other populations
Generalizability to other populations: In particular, discuss generalizability to a general Internet population, outside of a RCT setting, and general patient population, including applicability of the study results for other organizations

	1	2	3	4	5	
subitem not at all important	○	○	○	○	◉	essential

Clear selection

Does your paper address subitem 21-i?
Copy and paste relevant sections from the manuscript (include quotes in quotation marks "like this" to indicate direct quotes from your manuscript), or elaborate on this item by providing additional information not in the ms, or briefly explain why the item is not applicable/relevant for your study

No procedures were put in place to recruit a heterogenous sample based on sociodemographic characteristics, limiting the generalizability of the results. Participants may have been more interested in changing their PA behaviour (and thus more motivated to fulfill intervention requirements) and/or more competent with technology than women in the general population; they were also well-educated. These factors could have influenced their responses and limit the applicability of the results.

21-ii) Discuss if there were elements in the RCT that would be different in a routine application setting

Discuss if there were elements in the RCT that would be different in a routine application setting (e.g., prompts/reminders, more human involvement, training sessions or other co-interventions) and what impact the omission of these elements could have on use, adoption, or outcomes if the intervention is applied outside of a RCT setting.

	1	2	3	4	5	
subitem not at all important	◉	○	○	○	○	essential

Clear selection

Does your paper address subitem 21-ii?

Copy and paste relevant sections from the manuscript (include quotes in quotation marks "like this" to indicate direct quotes from your manuscript), or elaborate on this item by providing additional information not in the ms, or briefly explain why the item is not applicable/relevant for your study

Subitem 21-ii is not applicable. Only further research will determine if there are elements that would be different in a routine application setting.

OTHER INFORMATION

23) Registration number and name of trial registry

Does your paper address CONSORT subitem 23? *

Copy and paste relevant sections from the manuscript (include quotes in quotation marks "like this" to indicate direct quotes from your manuscript), or elaborate on this item by providing additional information not in the ms, or briefly explain why the item is not applicable/relevant for your study

This trial was registered at ClinicalTrials.gov (ID: NCT03601663, Date: July 26, 2018; http://clinicaltrials.gov/ct2/show/NCT03601663).

24) Where the full trial protocol can be accessed, if available

Does your paper address CONSORT subitem 24? *

Cite a Multimedia Appendix, other reference, or copy and paste relevant sections from the manuscript (include quotes in quotation marks "like this" to indicate direct quotes from your manuscript), or elaborate on this item by providing additional information not in the ms, or briefly explain why the item is not applicable/relevant for your study

Black M, Brunet J. Exploring the effect of an eHealth intervention on women's physical activity: Design and rationale for a randomized controlled trial. Digit Health. 2022;8:20552076221093134.

25) Sources of funding and other support (such as supply of drugs), role of funders

Does your paper address CONSORT subitem 25? *

Copy and paste relevant sections from the manuscript (include quotes in quotation marks "like this" to indicate direct quotes from your manuscript), or elaborate on this item by providing additional information not in the ms, or briefly explain why the item is not applicable/relevant for your study

The authors have no specific grant for this research from any funding agency in the public, commercial or not-for-profit sectors to declare. The role of funders is not applicable.

X27) Conflicts of Interest (not a CONSORT item)

X27-i) State the relation of the study team towards the system being evaluated

In addition to the usual declaration of interests (financial or otherwise), also state the relation of the study team towards the system being evaluated, i.e., state if the authors/evaluators are distinct from or identical with the developers/sponsors of the intervention.

	1	2	3	4	5	
subitem not at all important	○	○	○	○	◉	essential

Clear selection

Does your paper address subitem X27-i?

Copy and paste relevant sections from the manuscript (include quotes in quotation marks "like this" to indicate direct quotes from your manuscript), or elaborate on this item by providing additional information not in the ms, or briefly explain why the item is not applicable/relevant for your study

Competing interests
All authors declare that they have no competing interests.

About the CONSORT EHEALTH checklist

As a result of using this checklist, did you make changes in your manuscript? *

○ yes, major changes
○ yes, minor changes
◉ no

What were the most important changes you made as a result of using this checklist?

Your answer

How much time did you spend on going through the checklist INCLUDING making changes in your manuscript *

8 hours were spent on going through the checklist.

As a result of using this checklist, do you think your manuscript has improved? *

○ yes
◉ no
○ Other:

Would you like to become involved in the CONSORT EHEALTH group?

This would involve for example becoming involved in participating in a workshop and writing an "Explanation and Elaboration" document

○ yes
◉ no
○ Other:

Clear selection

Any other comments or questions on CONSORT EHEALTH

Your answer

STOP - Save this form as PDF before you click submit

To generate a record that you filled in this form, we recommend to generate a PDF of this page (on a Mac, simply select "print" and then select "print as PDF") before you submit it.

When you submit your (revised) paper to JMIR, please upload the PDF as supplementary file.

Don't worry if some text in the textboxes is cut off, as we still have the complete information in our database. Thank you!

Final step: Click submit !

Click submit so we have your answers in our database!

Submit Clear form

Never submit passwords through Google Forms.

This content is neither created nor endorsed by Google. Report Abuse - Terms of Service - Privacy Policy

Google Forms
